# Integrative multi-omics analysis reveals the genetic architecture of floral traits in *Anthurium*

**DOI:** 10.1093/hr/uhaf316

**Published:** 2025-11-08

**Authors:** Shengnan Lin, Chao Song, Dan Peng, Yaru Wang, Xiaoni Zhang, Yingxue Yang, Minlong Jia, Qingyun Leng, Shisong Xu, Xing’e Lin, Haiyan Li, Jinping Lu, Chengcheng Zhou, Xiao Wan, Jianrong Sun, Luke R Tembrock, Junmei Yin, Danqing Tian, Zhiqiang Wu, Junhai Niu

**Affiliations:** National Key Laboratory for Tropical Crop Breeding, Key Laboratory of Gene Resources and Germplasm Enhancement in Southern China, MARA, Tropical Crops Genetic Resources Institute, Chinese Academy of Tropical Agricultural Sciences, Haikou 571101, China; Shenzhen Branch, Guangdong Laboratory for Lingnan Modern Agriculture, Genome Analysis Laboratory of the Ministry of Agriculture, Agricultural Genomics Institute at Shenzhen, Chinese Academy of Agricultural Sciences, Shenzhen 518000, China; Kunpeng Institute of Modern Agriculture at Foshan, Shenzhen Branch, Guangdong Laboratory of Lingnan Modern Agriculture, Agricultural Genomics Institute at Shenzhen, Chinese Academy of Agricultural Sciences, Shenzhen 518124, China; Shenzhen Branch, Guangdong Laboratory for Lingnan Modern Agriculture, Genome Analysis Laboratory of the Ministry of Agriculture, Agricultural Genomics Institute at Shenzhen, Chinese Academy of Agricultural Sciences, Shenzhen 518000, China; Kunpeng Institute of Modern Agriculture at Foshan, Shenzhen Branch, Guangdong Laboratory of Lingnan Modern Agriculture, Agricultural Genomics Institute at Shenzhen, Chinese Academy of Agricultural Sciences, Shenzhen 518124, China; Chongqing Key Laboratory for Germplasm Innovation of Special Aromatic Spice Plants, College of Smart Agriculture, Chongqing University of Arts and Sciences, Chongqing 402160, China; Shenzhen Branch, Guangdong Laboratory for Lingnan Modern Agriculture, Genome Analysis Laboratory of the Ministry of Agriculture, Agricultural Genomics Institute at Shenzhen, Chinese Academy of Agricultural Sciences, Shenzhen 518000, China; School of Ecology, Hainan University, Haikou 570228, China; National Key Laboratory for Tropical Crop Breeding, Key Laboratory of Gene Resources and Germplasm Enhancement in Southern China, MARA, Tropical Crops Genetic Resources Institute, Chinese Academy of Tropical Agricultural Sciences, Haikou 571101, China; Shenzhen Branch, Guangdong Laboratory for Lingnan Modern Agriculture, Genome Analysis Laboratory of the Ministry of Agriculture, Agricultural Genomics Institute at Shenzhen, Chinese Academy of Agricultural Sciences, Shenzhen 518000, China; Kunpeng Institute of Modern Agriculture at Foshan, Shenzhen Branch, Guangdong Laboratory of Lingnan Modern Agriculture, Agricultural Genomics Institute at Shenzhen, Chinese Academy of Agricultural Sciences, Shenzhen 518124, China; Shenzhen Branch, Guangdong Laboratory for Lingnan Modern Agriculture, Genome Analysis Laboratory of the Ministry of Agriculture, Agricultural Genomics Institute at Shenzhen, Chinese Academy of Agricultural Sciences, Shenzhen 518000, China; Kunpeng Institute of Modern Agriculture at Foshan, Shenzhen Branch, Guangdong Laboratory of Lingnan Modern Agriculture, Agricultural Genomics Institute at Shenzhen, Chinese Academy of Agricultural Sciences, Shenzhen 518124, China; College of Horticulture, Shanxi Agricultural University, Taiyuan 030031, China; National Key Laboratory for Tropical Crop Breeding, Key Laboratory of Gene Resources and Germplasm Enhancement in Southern China, MARA, Tropical Crops Genetic Resources Institute, Chinese Academy of Tropical Agricultural Sciences, Haikou 571101, China; National Key Laboratory for Tropical Crop Breeding, Key Laboratory of Gene Resources and Germplasm Enhancement in Southern China, MARA, Tropical Crops Genetic Resources Institute, Chinese Academy of Tropical Agricultural Sciences, Haikou 571101, China; National Key Laboratory for Tropical Crop Breeding, Key Laboratory of Gene Resources and Germplasm Enhancement in Southern China, MARA, Tropical Crops Genetic Resources Institute, Chinese Academy of Tropical Agricultural Sciences, Haikou 571101, China; National Key Laboratory for Tropical Crop Breeding, Key Laboratory of Gene Resources and Germplasm Enhancement in Southern China, MARA, Tropical Crops Genetic Resources Institute, Chinese Academy of Tropical Agricultural Sciences, Haikou 571101, China; National Key Laboratory for Tropical Crop Breeding, Key Laboratory of Gene Resources and Germplasm Enhancement in Southern China, MARA, Tropical Crops Genetic Resources Institute, Chinese Academy of Tropical Agricultural Sciences, Haikou 571101, China; National Key Laboratory for Tropical Crop Breeding, Key Laboratory of Gene Resources and Germplasm Enhancement in Southern China, MARA, Tropical Crops Genetic Resources Institute, Chinese Academy of Tropical Agricultural Sciences, Haikou 571101, China; Zhejiang Institute of Landscape Plants and Flowers, Zhejiang Academy of Agricultural Sciences, Hangzhou, China; Taizhou Suzhong Horticulture Co., Ltd., Taizhou, China; Department of Agricultural Biology, Colorado State University, Fort Collins, CO 80525, USA; National Key Laboratory for Tropical Crop Breeding, Key Laboratory of Gene Resources and Germplasm Enhancement in Southern China, MARA, Tropical Crops Genetic Resources Institute, Chinese Academy of Tropical Agricultural Sciences, Haikou 571101, China; Sanya Research Institute, Chinese Academy of Tropical Agricultural Sciences, Sanya, China; The Engineering Technology Research Center of Tropical Ornamental Plant Germplasm Innovation and Utilization, Tropical Crops Genetic Resources Institute, Chinese Academy of Tropical Agricultural Sciences, Danzhou, China; Zhejiang Institute of Landscape Plants and Flowers, Zhejiang Academy of Agricultural Sciences, Hangzhou, China; National Key Laboratory for Tropical Crop Breeding, Key Laboratory of Gene Resources and Germplasm Enhancement in Southern China, MARA, Tropical Crops Genetic Resources Institute, Chinese Academy of Tropical Agricultural Sciences, Haikou 571101, China; Shenzhen Branch, Guangdong Laboratory for Lingnan Modern Agriculture, Genome Analysis Laboratory of the Ministry of Agriculture, Agricultural Genomics Institute at Shenzhen, Chinese Academy of Agricultural Sciences, Shenzhen 518000, China; Kunpeng Institute of Modern Agriculture at Foshan, Shenzhen Branch, Guangdong Laboratory of Lingnan Modern Agriculture, Agricultural Genomics Institute at Shenzhen, Chinese Academy of Agricultural Sciences, Shenzhen 518124, China; Zhejiang Institute of Landscape Plants and Flowers, Zhejiang Academy of Agricultural Sciences, Hangzhou, China; National Key Laboratory for Tropical Crop Breeding, Key Laboratory of Gene Resources and Germplasm Enhancement in Southern China, MARA, Tropical Crops Genetic Resources Institute, Chinese Academy of Tropical Agricultural Sciences, Haikou 571101, China; Sanya Research Institute, Chinese Academy of Tropical Agricultural Sciences, Sanya, China; The Engineering Technology Research Center of Tropical Ornamental Plant Germplasm Innovation and Utilization, Tropical Crops Genetic Resources Institute, Chinese Academy of Tropical Agricultural Sciences, Danzhou, China

## Abstract

*Anthurium*, a highly diverse genus in the family Araceae, is well known for its ornamental spathes and spadices. However, limited genomic resources hinder the study of floral traits and their evolutionary histories. Here, we present high-quality chromosome-level genome assemblies of *Anthurium andraeanum* and *Anthurium scherzerianum*. Comparative genomics revealed extensive chromosomal rearrangements and species-specific transposon expansions, which likely contributed to genome divergence. Two lineage-specific whole-genome duplications were identified, associated with gene family expansions linked to stress adaptation. Population structure analysis uncovered strong genetic admixture, reflecting widespread historical hybridization. Integrated transcriptomic and metabolomic analyses revealed dynamic regulatory networks governing spathe coloration through flavonoid–anthocyanin pathways. In addition, *CER3*, *KCS1*, and *KCS3* were identified as key regulators involved in wax biosynthesis. Notably, inflorescence evolution correlates with the loss of the floral identity genes *SOC1* and *AGL6*, highlighting conserved developmental pathways and lineage-specific innovations. Our findings provide foundational genomic resources for understanding *Anthurium* evolution, offer molecular targets for breeding programs, and elucidate transposon-driven genome expansion mechanisms that advance our knowledge of speciation in tropical epiphytes with exceptionally large genomes.

## Introduction

**Figure 1:**
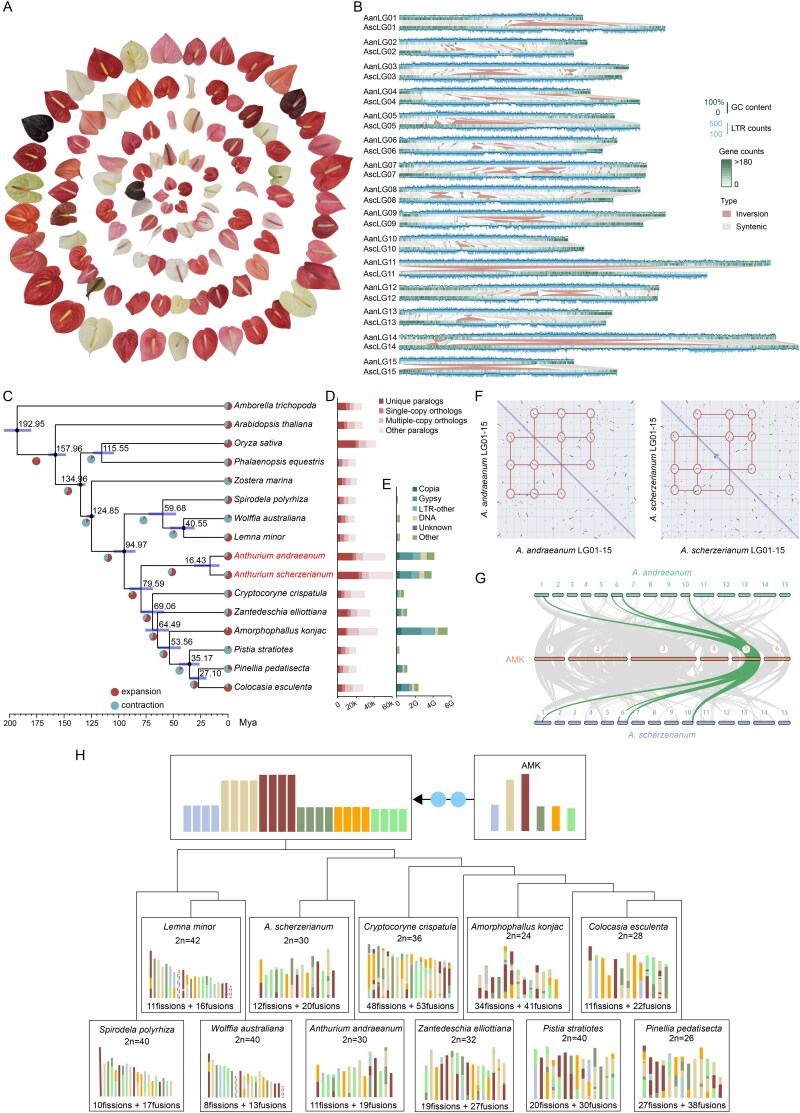
Comparative genomic analysis. (A) The diversity of spathes in *Anthurium*. (B) Genome features of *A. andraeanum* (Aan) and *A. scherzerianum* (Asc) genomes and structural variation (SV) distribution between the two *Anthurium* genomes. (C) Time-calibrated phylogeny, with node age and the 95% CIs labeled. Pie charts show the proportions of gene families that underwent expansion or contraction. Histograms show gene family categories and transposable element content. Black dots indicate the fossil calibration points used in the analysis. (D) Gene family classifications. (E) Comparison of repeat content across 11 Araceae species. The genomes of *Anthurium* spp. and *Amorphophallus konjac* exhibited substantially greater lengths of DNA transposons and LTR retrotransposons (Copia, Gypsy, and others) than the other species, correlating strongly with their larger genome sizes. (F) Intra-genome comparison within two *Anthurium* genomes. Macrosynteny patterns of two *Anthurium* genomes show that each region aligns with four syntenic regions in self. (G) Collinear comparison of two *Anthurium* genomes with the ancestral monocot karyotype (AMK). A clear 4:1 syntenic relationship supports the occurrence of two whole-genome duplication (WGD) events in both *Anthurium* species. (H) Evolutionary scenario of the Araceae genomes from the AMK. Chromosomes shown with dashed lines represent regions not covered by AMK. Comparative karyotype analysis reveals that Araceae genomes have undergone extensive chromosomal rearrangements, contributing to lineage-specific genome architectures.

*Anthurium* Schott., commonly known as the flamingo flower, is the most diverse and widespread genus in the ancient monocot family Araceae (order Alismatales). Native to the Neotropics, it spans from the Caribbean and Mexico to southern Brazil and Argentina, with an estimated 3000 species and over 1300 formally described species [1–4]. *Anthurium* species display diverse growth habits, including epiphytic, terrestrial, and climbing forms, and inhabit a wide range of tropical ecosystems [3, 5, 6]. They exhibit notable variation in vegetative and reproductive traits, such as leaf morphology, venation, inflorescence structure, and fruit color [4, 7–10]. The genus exhibits remarkable diversity in spathe morphology, a key trait affecting both its ecological adaptation and ornamental value (Fig. 1A). Among these species, *Anthurium andraeanum* and *A. scherzerianum* are the most iconic and economically important, prized for their colorful and long-lasting spathes. Extensively cultivated for decades, they rank second only to orchids in the tropical floriculture trade, significantly affecting the ornamental plant industry [11–13].

In *Anthurium* cultivars, the performance of spathes and spadices represents the most critical horticultural traits determining their ornamental quality and commercial value [14]. Spathe coloration is primarily regulated by anthocyanins. However, the biosynthetic pathways and regulatory mechanisms governing anthocyanin accumulation remain incompletely elucidated [15–18]. In addition, spathe longevity is closely linked to the composition of cuticular wax, which serves to protect tissues from intense solar radiation, reduce transpirational water loss, and thereby extend the flowering period [19–21]. Cuticular wax is mainly composed of long-chain fatty acids and their derivatives—such as alkanes, alcohols, and ketones—as well as terpenes, synthesized via fatty acid acyl reduction and decarboxylation reactions [22–25]. The spadix, with its vibrant color and unique structure, contrasts sharply with the spathe [26]. Composed of densely packed bisexual flowers lacking distinct petals, it varies in color from yellow, green, and white to pale purple, with some emitting a distinct fragrance [5, 27, 28]. Previous studies have demonstrated that the MADS-box gene family plays a central regulatory role in floral development, with the MIKC^C^ subfamily specifically implicated in floral organ differentiation as described by the ABCE model [29, 30]. This gene family has undergone substantial expansion through genome duplication events, facilitating functional divergence and innovation that drive floral morphological diversification and enhance plant adaptation to diverse ecological niches [30]. Despite this, the specific roles of MADS-box genes in spadix development in *Anthurium* remain poorly understood, and research addressing this is limited. Beyond its ornamental value, the spadix plays a crucial ecological role, enhancing pollination efficiency in Araceae by attracting insect and bird pollinators through its color and scent [31, 32]. However, the molecular mechanisms underlying the spatial and temporal regulation of these traits during spadix maturation remain largely unexplored.

The multi-omics analysis integrates genomics, transcriptomics, proteomics, metabolomics, epigenomics, and phenomics to capture the complexity of biological processes, leading to phenotypic manifestations [33–35]. The application of multi-omics analysis avoided the limitations of single-omics approach that only reflect fragmented biological information. Multi-omics analysis can help with the identification of key nodes and their regulatory relationships, revealing how genetic information flows and influences traits [36–38]. Our previous studies have shown that multi-omics analysis is an effective method for studying important plant traits [39, 40]. Hence, a comprehensive multi-omics approach is essential to uncover the genetic and biochemical foundations governing *Anthurium* floral characteristics.

High-quality genomes are vital for studying species evolution, adaptation, and genetic improvement, as they provide key insights into the origin, evolution, and gene expression of ornamental traits [41–45]. Over the past two decades, phylogenetic studies using both plastid and nuclear sequences have elucidated *Anthurium* evolution at the genus and species levels [2, 7, 46–50]. Although *Anthurium* is a monophyletic genus with recent divergence, its genomic resources have been limited until recently. The recent release of a chromosome-level genome assembly for *A. amnicola* [51] provides a valuable reference, yet functional genomics data for key ornamental traits such as the spathe and spadix remain scarce. Understanding the omics basis of these traits would accelerate breeding efforts, but further multi-omics studies are still needed.

In the current study, we performed *de novo* assembly of two high-quality *Anthurium* genomes and explored their evolutionary history and genetic variation. Here, 179 accessions were used and broadly grouped into four horticultural categories based on their primary commercial applications (e.g. cut flowers, potted flowers, ornamental foliage potted plants, and ornamental foliage Anthurium). While this classification reflects practical breeding and market distinctions, it may not accurately represent the underlying genetic structure or evolutionary relationships among accessions. Indeed, horticultural classification schemes often overlook critical biological factors, such as geographic origin, wild versus cultivated status, and species background, which are known to shape genetic diversity and adaptation in *Anthurium*. Acknowledging these limitations, our multi-omics analysis also serves to evaluate whether such application-based groupings are biologically meaningful or merely artificial constructs imposed by breeding goals. These findings provide new perspectives for understanding the complex relationship between the phenotype and genome of *Anthurium*. Moreover, by combining transcriptome and metabolome analyses, we elucidated the genetic basis of flower development, spathe coloration, and wax composition. This research not only uncovered the regulatory networks controlling flower color and development but also provided essential data for genetic breeding, offering valuable support for the scientific study and application of *Anthurium* and other ornamental plants.

## Results

### High-quality genome assembly and annotation of two *Anthurium* species

Two high-quality chromosome-level genomes for *Anthurium* were generated, containing 15 chromosomes with a total length of 4.15 Gb and a scaffold N50 value of 219.78 Mb for *A. andraeanum* ‘Alabama’ (*A. andraeanum*) and a total length of 3.84 Gb with a scaffold N50 value of 242.70 Mb for *A. scherzerianum* ‘Red Lantern’ (*A. scherzerianum*), consistent with the K-mer analysis (Table 1, Fig. 1B, and Figs S1–S3). The completeness of the assemblies was evaluated using 1416 conserved embryophyta proteins collected from the Benchmarking Universal Single-Copy Orthologs (BUSCO) program, showing 97.6% and 98.1% completeness for *A. andraeanum* and *A. scherzerianum*, respectively (Table S4 and Fig. S4). Both *A. andraeanum* and *A. scherzerianum* genomes exhibited high base accuracy, coverage, and assembly quality (Table S5). Hi-C chromatin contact maps showed strong intrachromosomal interactions and clear boundaries between chromosomes, confirming the accuracy and chromosome-scale continuity of the assemblies (Fig. S5A and B). In addition, comparative analyses showed that the Hi-C-based assembly was highly consistent with the high-density genetic map generated from the biparental segregating population of *A. andraeanum* (Fig. S5C and D). In summary, the two new assemblies exhibit excellent continuity, accuracy, and completeness.

**Table 1:** Basic information statistics of two *Anthurium* genomes

Genomic features	*A. andraeanum*	*A. scherzerianum*
Assembly size (Gb)	4.15	3.84
No. of contig	1,404	1,232
No. of scaffold	216	175
Contig N50 (Mb)	5.89	7.35
Scaffold N50 (Mb)	219.78	242.70
Genome complete BUSCO (%)	97.6	98.1
LAI	19.66	16.35
QV	55.93	60.68
Repeat sequences (%)	82.07	81.96
Predicted protein-coding genes	52,380	61,287
Average No. of exons per gene	3.5	3.7
Average coding sequence length (bp)	965	950
Average exon length (bp)	272	258
Average intron length (bp)	9,511	8,930
Protein complete BUSCO (%)	93.1	93.7

A total of 3.41 Gb and 3.15 Gb transposable element (TE) sequences were predicted in the *A. andraeanum* and *A. scherzerianum* genomes, accounting for 82.07% and 81.96% of the total assembly length, respectively (Table S6). Among the TEs, Gypsy elements were dominant, accounting for 42.06% of the average annotated TE content per assembly and 34.50% of the total number of assemblies (Table S6). By integrating *ab initio* prediction, homology-based, and transcriptome evidence-based predictions, 52,380 and 61,287 protein-coding genes were predicted in *A. andraeanum* and *A. scherzerianum*, covering 96.3% and 96.4% of the BUSCO genes, respectively (Table S7 and Fig. S4). On average, *A. scherzerianum* had a longer total gene length than that of *A. andraeanum*, whereas *A. andraeanum* had a longer average intron length (Table S7). In addition, we annotated 1,993 and 2,826 non-coding RNAs (ncRNAs) in the *A. andraeanum* and *A. scherzerianum* genomes, respectively, including 175 and 134 microRNAs (miRNAs), 653 and 1,030 transfer RNAs (tRNAs), 564 and 1,074 ribosomal RNAs (rRNAs), and 597 and 583 small nuclear RNAs (snRNAs) (Table S7). We also performed functional annotation of the protein-coding genes (Table S8).

### Evolution and comparative genomic analyses

Using *A. andraeanum* as the reference genome, we identified 39.51 million single nucleotide polymorphisms (SNPs) and 3.84 million small insertions and deletions (InDels, length < 50 bp) in *A. scherzerianum*, with chromosomes 13 and 3 harboring the highest numbers of SNPs and InDels per kilobase (Table S9). Among these variations, 715,808 SNPs and 52,666 InDels were located in coding sequence regions (Table S10). A total of 2,727,045 structural variations (SVs) were detected relative to the *A. andraeanum* genome, including 1,078,798 deletions, 1,564,762 insertions, 10,935 translocations, 725 inversions, and 71,825 duplications (Fig. 1B and Table S11). Notably, megabase-scale inversions and translocations were observed on most chromosomes, excluding chromosomes 2, 4, 6, 10, and 13, which showed more structural variation (Fig. 1B and Table S11). These extensive sequence and structural variations may contribute to the morphological, physiological, and ecological divergence observed between the two *Anthurium* species.

Maximum-likelihood phylogenetic trees with divergence time estimation were constructed based on 441 single-copy orthologous genes from 16 species. These included two *Anthurium* species generated in this study, and nine species from other genera in the families Araceae, as well as *Zostera marina*, *Phalaenopsis equestris*, *Oryza sativa*, *Arabidopsis thaliana*, and *Amborella trichopoda* as an outgroup. *Anthurium* diverged from its closest relatives ~79.59 million years ago (Mya), while the divergence time between *A. andraeanum* and *A. scherzerianum* was estimated at ~16.43 Mya (Fig. 1C). We identified 9,148 and 16,041 unique paralogs in *A. andraeanum* and *A. scherzerianum* genomes, respectively (Fig. 1D). The Kyoto Encyclopedia of Genes and Genomes (KEGG) enrichment results of unique paralogs showed that they were mainly distributed in ‘cutin, suberin, and wax biosynthesis’, ‘fatty acid biosynthesis’, ‘porphyrin and chlorophyll metabolism’, and ‘lipid metabolism’, suggesting the evolution of key enzyme genes associated with metabolite synthesis and pathways for environmental adaptation in *Anthurium* (Fig. S6). A total of 1,454 and 1,131 gene families were significantly expanded, and 594 and 611 gene families were contracted in the *A. andraeanum* and *A. scherzerianum* genomes, respectively (Fig. 1C). The expanded gene families were enriched in ‘phenylpropanoid biosynthesis’, ‘zeatin biosynthesis’, and ‘plant-pathogen interaction’, which are related to plant tolerance and stress resistance for adaptation to the environment in *Anthurium* (Fig. S7). By comparing the content of various repeats in the 11 Araceae species, we observed that the lengths of DNA transposons and LTR retrotransposons, including Copia, Gypsy, and other elements, in the two *Anthurium* and *Amorphophallus konjac* genomes were much greater than those in the other eight Araceae species, which strongly correlated with genome size expansion (Fig. 1E and Table S6). Analysis of LTR insertion times in the two *Anthurium* genomes revealed distinct bursts of Copia (*A. andraeanum*: 18 kya and *A. scherzerianum*: 16 kya) and Gypsy (*A. andraeanum*: 20 kya and *A. scherzerianum*: 8 kya) elements across the two lineages (Fig. S8). Copia expansion events in *A. andraeanum* and *A. scherzerianum* occurred within a narrow timeframe, whereas Gypsy activity exhibited marked divergence between lineages. These findings suggest that lineage-specific regulatory mechanisms or environmental pressures shape TE dynamics.

Whole-genome duplication (WGD) events are widely recognized as a major driver of speciation, supplying abundant genetic material that promotes species diversification and enhances environmental adaptability, thereby playing a pivotal role in plant evolutionary processes [52, 53]. The identification of conserved synteny blocks is fundamental for inferring WGD events [54]. Intragenomic collinearity analysis identified extensive duplicated blocks within each genome, reinforcing the hypothesis of two WGDs in *A. andraeanum* and *A. scherzerianum* (Fig. 1F). A comparison with the reconstructed ancestral monocot karyotype (AMK) [55] revealed a clear 4:1 synteny relationship, providing strong support for two WGD events in both *Anthurium* species (Fig. 1G, Fig. S9, and Tables S12 and S13). Ks distribution analysis revealed a single peak related to ancient WGD events (Fig. S10). Although the Ks distribution does not show two clearly separated peaks, this pattern is consistent with previous reports in other Araceae species, where closely spaced or overlapping WGD events also resulted in a single Ks peak [56–60]. These observations collectively support the occurrence of two WGD events in the Araceae lineage, though they may be difficult to resolve using Ks distributions alone. In addition, chromosome rearrangement events were considered an important factor in genome evolution and speciation [61, 62]. Based on AMK, the evolutionary trajectories of the 14 selected species in the Araceae family revealed that the karyotype of the Lemnoideae subfamily has remained relatively stable (Fig. S11). In contrast, the remaining Araceae species have undergone complex chromosomal fusions and structural rearrangements, leading to a highly diverse range of chromosome numbers (2*n* = 24–40) and karyotypic patterns among extant species (Fig. 1H). Karyotype evolution analysis of Araceae species based on AMK provides insights into how chromosomal variations have driven species diversification within the family.

### Population structure analyses

**Figure 2:**
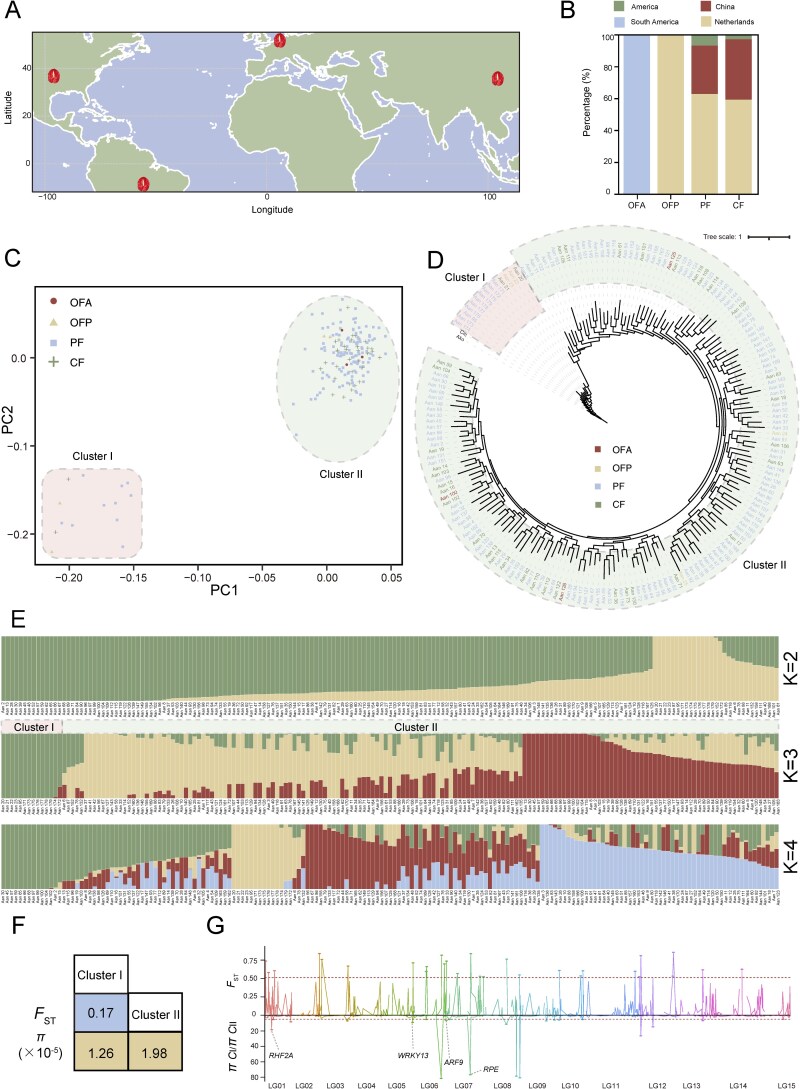
Geographic distribution, genetic diversity, and population structure of *Anthurium* accessions. (A) Global distribution of the 179 *Anthurium* accessions used in this study, showing their geographical origins. (B) Proportion of different geographical regions within each horticultural group—CF (cut flowers), PF (potted flowers), OFP (ornamental foliage potted plants), and OFA (ornamental foliage Anthurium). (C) Principal component analysis (PCA) of 179 *Anthurium* accessions based on high-quality SNPs, revealing two major genetic clusters. (D) Maximum likelihood phylogeny with 1,000 bootstrap replicates showing two main clades. Bootstrap values from 1,000 replicates are shown at each node, indicating the level of branch support. Accessions cluster into two major clades (Cluster I and Cluster II), consistent with PCA (panel C) and population structure analysis (panel E). (E) Population structure analysis of the 179 *Anthurium* accessions based on the SNP dataset. Each vertical bar represents one accession, with segments indicating the proportion of genetic components assigned to different clusters, consistent with the phylogenetic and PCA results. (F) Mean nucleotide diversity (*π*) in each cluster and population differentiation (*F*_ST_) between clusters. (G) Genome-wide screening of selective sweeps in Cluster I versus Cluster II *Anthurium* accessions. The red dotted lines represent the threshold of the top 5% *F_ST_* and 5% ratio of *π*  _CI_ (Cluster I) to *π*  _CII_ (Cluster II), respectively.

To better understand the dynamic genomic variation and diversity within diversified individuals, a total of 179 *Anthurium* accessions, categorized into four categories—cut flowers (CF), potted flowers (PF), ornamental foliage potted plants (OFP), and ornamental foliage Anthurium (OFA)—were selected based on their horticultural applications and geographical distribution (Fig. 2A and B, Table S14). A total of 2.26 billion reads were generated from Specific-Locus Amplified Fragment sequencing (SLAF-seq), and the average base call accuracy reached Q30 in 94.70% of sequences, with an average GC composition of 41.31%. The clean reads were aligned to the *A. andraeanum* reference genome using BWA software. The mapping rates of individuals to the reference genome ranged from 80.33% to 99.41%, indicating a high genetic similarity between the 179 *Anthurium* accessions and the *A. andraeanum* genome. Among them, ‘Choco’ from the Netherlands showed the highest mapping rate (99.41%), while H22254 from Xishuangbanna (China) is the accession with the lowest mapping rate (80.33%) (Table S14). Subsequently, we identified 14,006,947 high-quality SNPs, and 1,055 4DTv sites were screened from this SNP dataset and used for subsequent population genetics analysis.

The principal component analysis (PCA) of high-quality SNPs revealed that the 179 *Anthurium* accessions clustered into two major genetic groups: Cluster I (comprising CF, PF, and OFA accessions) and Cluster II (including CF, PF, OFA, and OFP) (Fig. 2C). Phylogenetic analysis based on the same SNP dataset yielded a consistent classification, dividing accessions into two main clades (Fig. 2C and D, Fig. S12A). Furthermore, population structure analysis at *K* = 3 corroborated this division, clearly delineating the two groups in agreement with PCA and phylogenetic results (Fig. 2C–E and Fig. S12B). Pairwise genome-wide *F*_ST_ between Cluster I and Cluster II was estimated at 0.17, indicating moderate genetic differentiation. Nucleotide diversity (*π*) was 1.26 × 10^−5^ in Cluster I and 1.98 × 10^−5^ in Cluster II, indicating that both groups maintain substantial within-group genetic variation (Fig. 2F). Consistent with these results, the SNP-based clustering did not fully support the traditional horticultural groupings of CF, PF, OFP, and OFA, suggesting that these categories represent artificial classifications that only partially reflect the underlying genetic structure.

By comparing the two clusters, the top 5% of regions based on the *π*  _CI_ (Cluster I) to *π*  _CII_ (Cluster II) ratio were identified as selective sweep hotspots, which intersected with gene annotations to yield 26 candidate genes, most of which have unknown functions. Notably, four genes have been well studied: *AanV1_01G014960* (*RHF2A*), involved in gametogenesis and post-meiotic mitosis; *AanV1_05G028290* (*WRKY13*), regulating stem development and enhancing cadmium tolerance; *AanV1_06G027080* (*ARF19*), regulating auxin- and ethylene-responsive gene expression, thereby controlling root and stem development; and *AanV1_07G016990* (*RPE*), a key enzyme in the Calvin–Benson cycle affecting photosynthetic capacity. Similarly, *F*_ST_ analysis identified the top 5% of regions as differentiation hotspots, yielding 16 candidate genes with unknown functions. Together, these candidate genes in hotspot regions may provide a molecular basis for trait formation and adaptive evolution in *Anthurium*.

### Regulatory dynamics of inflorescence development in Araceae

**Figure 3:**
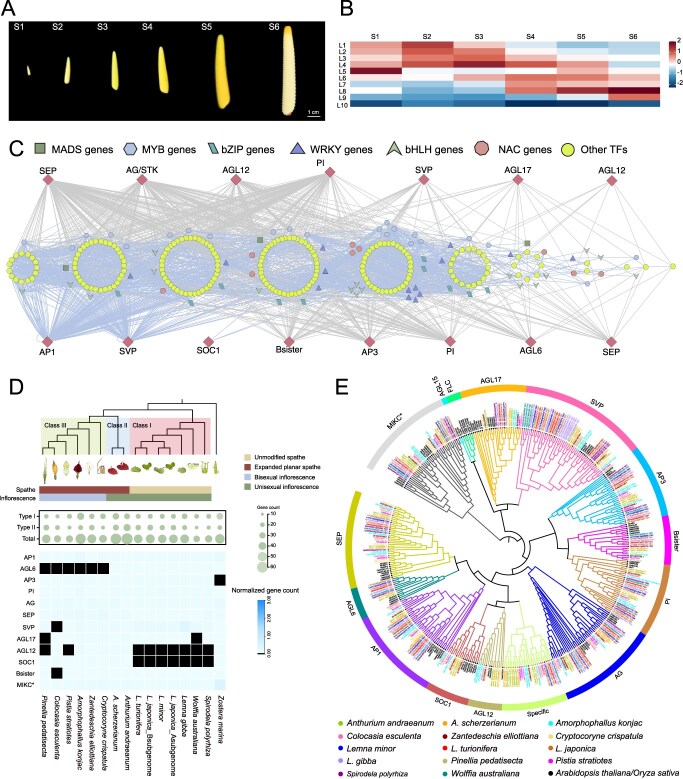
Dynamics of inflorescence development regulation in *Anthurium*. (A) The six spadix developmental stages (S1–S6) of *A. andraeanum*. (B) Heatmaps of average TPMs (z-score normalized) at each level of TO-GCN at each spadix developmental point in *A. andraeanum*. (C) Predicted regulatory network and the connection among TFs and MADS-box genes involved in flower development pathways. L1 to L10 indicate the levels identified in the ordered gene co-expression network. (D) Comparison of inflorescence morphology and normalized gene copy numbers in Araceae species. The normalization on the family dataset divides the gene count number of each species by the largest gene copy number within that family. *L. japonica* genome consists of A and B subgenomes. (E) Maximum likelihood phylogenetic tree of MADS-box gene family in Araceae species.

To analyze the regulation of spadix development in *A. andraeanum*, differentially expressed genes (DEGs) between any two stages were fed into the time-ordered gene co-expression networks (TO-GCN) analysis (Fig. 3A). The major GCN comprised 10 time-ordered levels (denoted L1 to L10 in Fig. 3B), matching the order of expression time of the transcription factor (TF) genes cross six flowering stages (S1–S6). A total of 10,178 genes (650 TFs and 9,528 structural genes) were found, with an average transcript per million (TPM) > 0.5 and exhibiting significant differentiation between any two samples among the six flowering times. As the initial node, the MADS-box TF *APETALA1* (*AP1*), which is highly expressed at the first time point but not later, was selected to generate the TO-GCN. TO-GCN revealed a co-expression network involving 650 differentially expressed TFs and 15 genes in the ABC model of floral development (Fig. 3C). The general pattern revealed that most network members (166 TFs) appeared at S1–S3, 82 TFs at S4–S5, and 28 TFs at S6 (Fig. 3B and C).

To understand the evolutionary basis of *Anthurium* inflorescences in comparison with those of other species, we selected 14 Araceae species (including the two *Anthurium* species in the present study) and one closely related species (*Z. marina*) for further analysis. Based on spathe and inflorescence morphology, these 14 Araceae species could be divided into three classes (Fig. 3D). Class I, characterized by unmodified spathes and unisexual inflorescences, includes *Spirodela polyrhiza*, *Wolffia australiana*, and species from the genus *Lemna*. Class II, which possesses an expanded planar spathe and an unsexual inflorescence, includes *A. andraeanum* and *A. scherzerianum*. Class III, characterized by an expanded planar spathe and bisexual flower inflorescences, includes *Cryptocoryne crispatula*, *Zantedeschia elliottiana*, *A. konjac*, *Pistia stratiotes*, *C. escuienta*, and *Pinellia pedatisecta*. In addition, we constructed a phylogenetic tree based on the protein sequences of 14 Araceae species and observed a grouping pattern similar to the aforementioned types (Fig. 3D). By analyzing the copy numbers of MADS-box genes across 15 species, species-specific loss, expansion, and contraction were identified (Fig. 3D and Table S15). We found that the *SUPPRESSOR OF OVEREXPRESSION OF CO1* (*SOC1*) homolog was absent in Class I. Conversely, the E-function *AGAMOUS-Like 6* (*AGL6*) homolog was missing in Class III. Notably, the two *Anthurium* species retained both the *SOC1* and *AGL6* homologs. Phylogenetic analysis of type II MADS-box genes revealed the presence of a distinct clade alongside 13 established subgroups referred to as species-specific genes (it belongs to the type II but does not fall into any of the 13 established subgroups). These genes were found in 13 Araceae species, except *S. polyrhiza*. Notably, the two examined *Anthurium* species harbored the highest number of species-specific genes (Fig. 3E, Fig. S13, and Table S16). This may be associated with their intermediate evolutionary position in the diversification of floral morphology, underscoring their unique status within the evolutionary trajectory of Araceae.

### The regulatory network for spathe color formation in *Anthurium*

**Figure 4:**
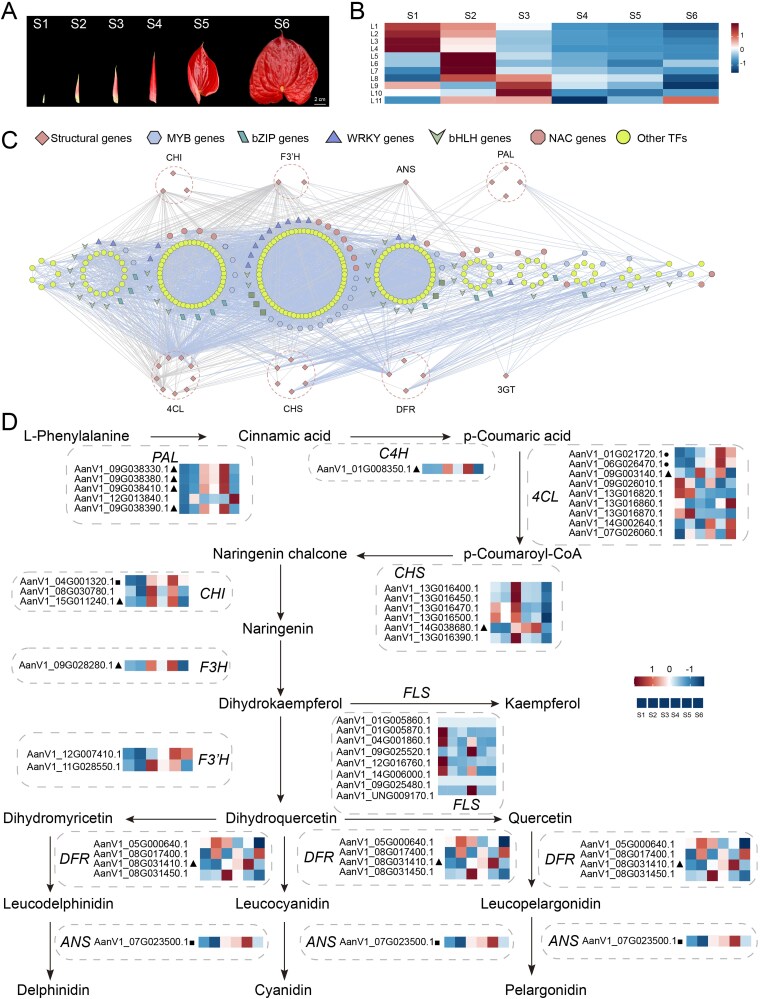
Dynamics of spathe pigmentation regulation in *Anthurium*. (A) The six spathe developmental stages (S1–S6) of *A. andraeanum*. (B) Heatmaps of average TPM (z-score normalized) at each level of TO-GCN at each spathe developmental point in *A. andraeanum*. (C) Predicted regulatory network and the connection among TFs and structural genes involved in anthocyanin/flavonol biosynthesis pathways. L1 to L11 indicate the levels identified in the ordered gene co-expression network. (D) The anthocyanin/flavonol biosynthesis pathways in *A. andraeanum*. Genes significantly positively correlated with anthocyanin and/or flavonoid content (pearson correlation coefficient > 0.8, p < 0.05) are indicated by different symbols: circles for anthocyanin, triangles for flavonoid, and squares for both.

Spathe color is a key ornamental trait of *Anthurium*. To investigate the regulatory network underlying spathe pigmentation, we identified key genes involved in flavonoid biosynthesis, particularly anthocyanins, and analyzed their expression patterns along with anthocyanin accumulation throughout spathe development (Fig. 4A). During spathe development, the flavonoid content exhibited a biphasic oscillatory pattern, decreasing initially from stages S1 to S3 before rising progressively through S5, followed by a marked decline to its nadir at S6 (Fig. S14A). In contrast, anthocyanin accumulation demonstrated a unidirectional trajectory, increasing steadily across developmental stages and peaking at S5, which temporally correlated with full spathe coloration (Fig. S14B). We performed time-ordered comparative transcriptome analyses to explore the regulatory networks influencing pigment deposition during spathe development. Based on the expression patterns associated with spathe pigmentation, the time-ordered sub-networks could be assigned to four major patterns: high-level expression of L1–L4 at S1, L5–L8 at S2, L9–L11 at S3, and generally low expression for L1–L11 during S4–S6 stages (Fig. 4B). During the S1–S3 stages, numerous TFs, including ERF, bHLH, bZIP, GRAS, MADS, MYB, NAC, and WRKY, were highly expressed (Fig. 4C). This suggests that the genes associated with flavonoid and anthocyanin biosynthesis are predominantly activated during these stages and play active regulatory roles. In contrast, during the S4–S6 stages, the number of actively transcribed TF genes declined significantly, and only a few families, such as bHLH, MYB, TCP, and NAC, remained active (Fig. 4C).

In addition, all genes related to flavonoid and anthocyanin biosynthesis were identified based on the high-quality genome assembly of *A. andraeanum*. We identified 33 enzyme genes with predicted functions in anthocyanin and flavonoid biosynthesis (Fig. 4D). Pearson correlation analysis was performed to assess the relationships between the expression profiles of key enzyme genes and anthocyanin and flavonoid contents. With the exception of *Flavonol Synthase* (*FLS*), at least one copy of each remaining key enzyme gene was significantly positively correlated with anthocyanin or flavonoid content (correlation coefficient > 0.8, *P* < .05; Fig. 4D). These results support the positive regulatory role of these genes in flavonoid biosynthesis. In addition, TO-GCN was constructed based on the gene expression patterns (L1–L11) of *A. andraeanum* during spathe development (S1–S6) (Fig. 4C), highlighting significant correlations between 33 enzyme genes and TFs. The correlation patterns showed that the associations between the enzyme genes and TFs were mainly distributed in L1–L6, mostly involving the enzyme genes *chalcone isomerase* (*CHI*), *flavonoid 3′-hydroxylase* (*F3′H*), *anthocyanidin synthase* (*ANS*), *4-coumarate-CoA ligase* (*4CL*), *chalcone synthase* (*CHS*), and *dihydroflavonol reductase* (*DFR*) (Fig. 4C). Overall, these regulatory networks provide a reference for further analyses of the molecular mechanisms underlying spathe spatial pigmentation in *A. andraeanum*.

### Metabolite profiling reveals the color diversity of spathes driven by different metabolite groups

**Figure 5:**
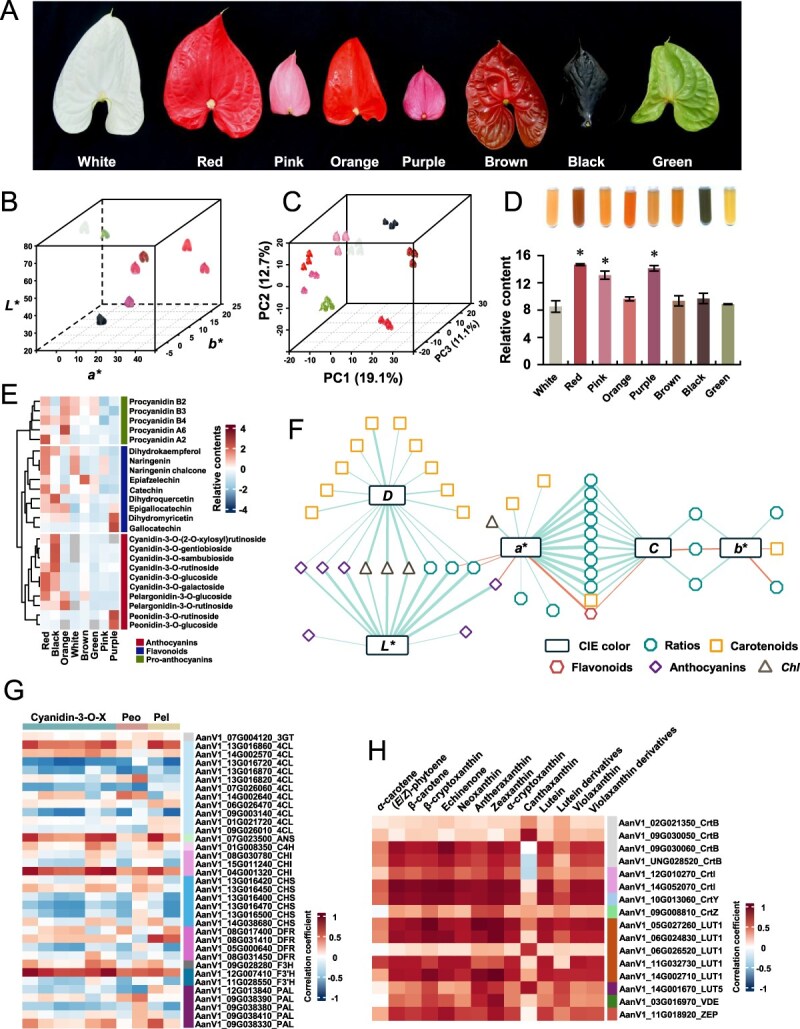
Metabolite profiling reveals spathe color formation by different metabolite groups. (A) Image of bracts from different cultivars. (B) 3D scatter plot of the color differences in *Lab* color space. (C) PCA plot of non-targeted metabolite profiling. (D) Total flavonoids extracted from the spathes of eight cultivars. (E) Relative content of anthocyanins, flavonoids, and pro-anthocyanins. The * indicates the significant differences (p < 0.0001) between white and the other cultivars using Dunnett test. (F) Correlation-based network between Lab color traits and different metabolite groups. Details of the correlations are listed in Tables S15 and S16. (G) Heatmap of correlations between candidate genes and anthocyanins. Cyanidin-3-O-X, Peo, and Pel represent the cyanidins, peonidins, and pelargonidins shown in (E), respectively. (H) Heatmap of correlations between candidate genes and carotenoids.

To elucidate light on the mechanism underlying color regulation in *Anthurium* cultivars, we examined eight cultivars with different spathe colors (Fig. 5A). Color values were quantified and visualized in a scatter plot, presenting color variations between different cultivars (Fig. 5B). We then conducted a metabolomic analysis to investigate the potential relationship between spathe color and metabolite composition. Clustering based on metabolite profiles revealed that white and pink spathes grouped closely together, whereas a similar close association was observed between orange and purple spathes (Fig. 5C). In addition, we calculated the total flavonoid content and found that red, pink, and purple spathes possessed significantly higher flavonoid contents with the white spathe as a reference (Fig. 5D). Using non-targeted metabolite profiling, we identified 10 anthocyanins belonging to pelargonidins (pelargonidin derivatives) and cyanidins (cyanidin and peonidin derivatives) (Fig. 5E). However, only four anthocyanins were identified in all cultivars. Red and black spathes accumulated higher contents of most anthocyanins (8 out of 10), whereas the white, green, and pink spathes had relatively lower contents of the examined anthocyanins. The flavonoids in the middle heatmap presented a pattern similar to that of the anthocyanins (Fig. 5E). We further correlated the parameters of spathe colors with the metabolite profiles to explore the possible mechanisms of spathe color formation. The correlation-based network revealed that L*a*b* values were negatively associated with the ratios between different metabolite groups (Fig. 5F and Table S17). For instance, a* correlated with 14 ratio parameters, including the ratios of carotenoids to anthocyanins, carotenoids to flavonoids, anthocyanins to flavonoids, and the sum of carotenoids and anthocyanins to flavonoids. In addition to a*, b* was correlated only with ratio-related parameters. The *D* parameter correlated with most metabolite classes of carotenoids. These results indicate that the sensory color of the spathe may be resulted from a complex regulation of different metabolite classes.

As anthocyanins and carotenoids were identified in non-targeted metabolite profiling, we screened the candidate genes responsible for anthocyanin and carotenoid biosynthesis (Fig. 5G and H). The heatmap of correlations highlighted the candidate genes with relative strong associations across anthocyanins (Fig. 5G), showing that *AanV1_07G004120*, *AanV1_13G016860*, *AanV1_07G023500*, *AanV1_01G008350*, *AanV1_04G001320*, *AanV1_13G016450*, *AanV1_08G031410*, *AanV1_09G028280*, *AanV1_12G007410*, and *AanV1_09G038330* were the candidate genes for *3-O-Glycosyltransferase* (*3GT*), *4CL*, *ANS*, *C4H*, *CHI*, *CHS*, *DFR*, *F3H*, *F3′H*, and *Phenylalanine Ammonia-Lyase* (*PAL*), respectively. With respect to the correlation pattern, more than one candidate genes among the key genes were involved in carotenoid biosynthesis (Fig. 5H). For example, both *AanV1_09G030060* and *AanV1_UNG028520* of *Phytoene Synthase* (*CrtB*) were strongly correlated with carotenoids. A similar pattern to that of *CrtB* was observed for *Lutein-deficient 1* (*LUT1*) (Fig. 5H), indicating that further identification is needed to screen the candidate genes.

### Wax layer prolongs the vase life of *Anthurium* spathe as ornamental plants

**Figure 6:**
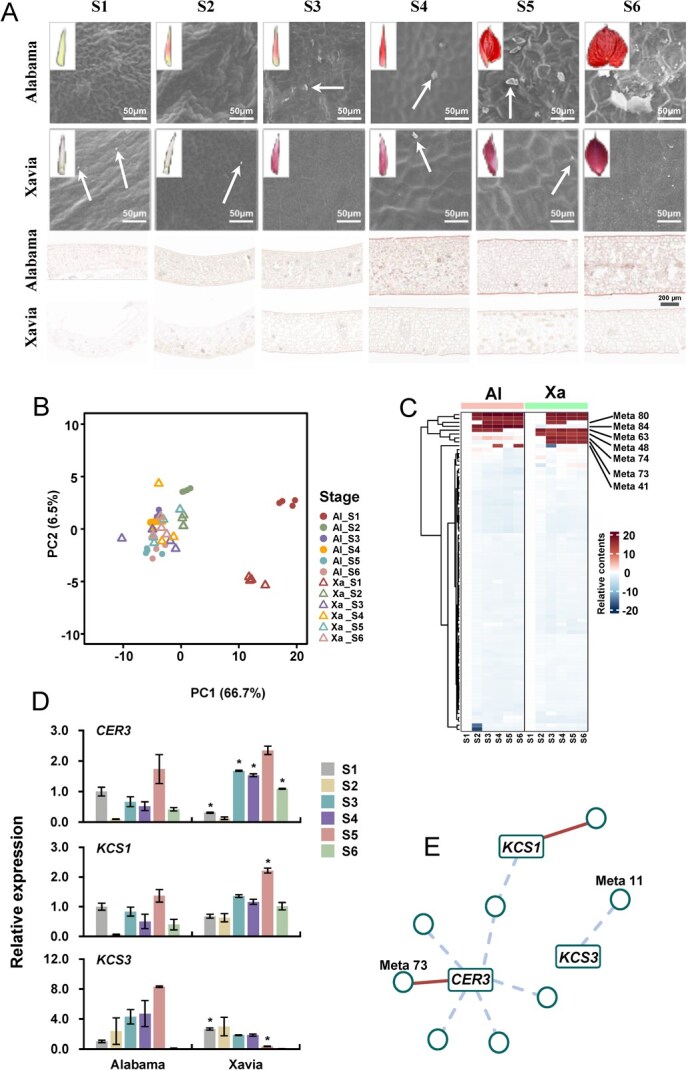
Cuticular waxes of ‘Alabama’ and ‘Xavia’ during spathe development. (A) Analysis of crystals on spathe surfaces (1^st^ and 2^nd^ rows) and wax layers on spathe cross-sections (3^rd^ and 4^th^ rows) using scanning electron microscopy and frozen sections, respectively. The arrows indicate wax crystals. The photograph on the top left corners showed the spathe at different developmental stages. (B) PCA plot of wax-related metabolites in the spathe of ‘Alabama’ (Al) and ‘Xavia’ (Xa) at different developmental stages. (C) Relative contents of wax-related metabolites in ‘Alabama’ (Al) and ‘Xavia’ (Xa) across different developmental stages. (D) Expression levels of candidate genes related to wax biosynthesis in the spathes of ‘Alabama’ and ‘Xavia’. The * indicates the significant difference (p < 0.05) between ‘Alabama’ and ‘Xavia’ at different developmental stages using t-test. (E) Correlation analysis between metabolites and candidate genes.

To explore the possible cuticular wax-regulating mechanisms that may enhance the vase life *Anthurium*, we selected two cultivars of *A. andraeanum*, ‘Alabama’, and ‘Xavia’, with contrasting vase lives. ‘Alabama’, which has a longer freshness lifetime, had a lighter spathe surface than did ‘Xavia’, indicating a difference in the cuticular wax of the spathe between the two cultivars. We used scanning electron microscopy to examine the cuticular wax crystals on the spathes of the two cultivars (Fig. 6A). Granular wax crystals were clearly observed, and their number considerably increased from S3 in ‘Alabama’. Granular wax crystals were sparsely observed on the epidermis of ‘Xavia’ (Fig. 6A, first and second rows). In addition to wax crystal number, the cross-section of wax crystals and spathes showed similar indications that after S3, ‘Alabama’ exhibited a clearer wax layer than that in ‘Xavia’ (Fig. 6A, third and fourth rows). To explore the mechanism underlying the wax differences between ‘Alabama’ and ‘Xavia’, we analyzed cuticular wax in the two cultivars and identified 82 metabolites associated with cuticular wax. Among these metabolites, alkanes were the major metabolite class (Table S19 and S20), indicating that the cuticular wax of *Anthurium* spathes mainly comprises alkanes. The PCA plot showed that the earlier stages (S1 and S2) of both varieties were clearly separated from the other four stages (Fig. 6B), reflecting differences in metabolite composition among developmental stages. Consistent with the cross-section observations of spathes (Fig. 6A), wax deposition appeared less prominent during the first two stages. In addition, the two cultivars may have presented similar wax profiles as the later stages (S3 and S4) remained close to each other. Focusing on the wax profiles, the two cultivars showed no differences in most metabolites with respect to accumulation trends (Fig. 6B). Notably, seven metabolites showed significant differences in content and accumulation patterns between the two cultivars across developmental stages, suggesting that these metabolites may play a role in the variations in cuticular wax between the two cultivars (Fig. 6C). We identified three genes (*CER3*, *KCS1*, and *KCS3*) involved in the biosynthesis and transport of waxes [63, 64] and obtained their expression levels in the two cultivars across developmental stages (Fig. 6D). The expression patterns of *ECERIFERUM 3* (*CER3*) and *β-Ketoacyl-CoA Synthase 1* (*KCS1*) genes were quite similar, whereas the obvious differences were observed in *β-Ketoacyl-CoA Synthase 3* (*KCS3*) genes in the spathes of the two cultivars, showing increased and decreased levels in ‘Alabama’ and ‘Xavia’, respectively (Fig. 6D). We further explored the association between the identified genes and wax profiles to address the possible mechanisms of cuticular wax formation (Fig. 6E). The correlation-based network revealed that three genes, *KCS1*, *KCS3*, and *CER3*, were significantly associated with several metabolites. The expression of *CER3* was positively associated with the content of Meta 73 and nonacosane (Fig. 6E) and significantly differed between ‘Alabama’ and ‘Xavia’ (Table S19).

## Discussion

In this study, we presented two chromosome-scale genome assemblies of the most well-known species of *Anthurium* spp. (*A. andraeanum* and *A. scherzerianum*) and perform a comprehensive comparative genomic analysis to elucidate their genomic divergence, evolutionary trajectories, and adaptive mechanisms. Population structure analyses revealed an intricate genetic admixture across *Anthurium* populations, driven by extensive historical hybridization. By integrating genomic, transcriptomic, and metabolomic datasets, we uncovered the genetic underpinnings of key floral traits, particularly the evolution of the spadix and diversification of spathe colors.

Comparative genomic analyses revealed substantial structural divergence between the two *Anthurium* species, marked by a large-scale chromosomal rearrangement. Gene family analysis identified lineage-specific expansions, particularly in gene families associated with phenylpropanoid biosynthesis, stress response, and pathogen defense, which likely provide a molecular basis for ecological adaptation in diverse tropical environments. Additionally, TE activity, especially within LTR retrotransposons, such as Gypsy and Copia, played a key role in genome size variation and structural evolution. Copia elements underwent a relatively synchronous amplification, while Gypsy elements exhibited more dispersed expansion timelines. These TE amplification events coincide temporally with the dramatic climatic shifts during the last glacial period, indicating a potential association. However, it is important to note that temporal correlation alone does not confirm an adaptive role for TE activation. Further functional validation is necessary to determine whether TE-mediated genomic plasticity contributed to post-glacial environmental adaptation. Nonetheless, such genomic flexibility could theoretically facilitate rapid adaptation to environmental changes and potentially drive phenotypic divergence, which may accelerate speciation and trait diversification in *Anthurium*. Two closely timed WGD events were identified, consistent with patterns observed in other Araceae species [56–60]. While these shared WGDs reflect conserved evolutionary mechanisms, *Anthurium* genomes display unique trajectories of accelerated structural reorganization and TE proliferation, paralleling genome expansion in *A. konjac* but contrasting with the streamlined genome of *S. polyrhiza* [56, 59]. These findings underscore how conserved polyploidization and lineage-specific genomic innovations jointly shape ecological adaptations in Araceae, advancing comparative frameworks for studying evolutionary drivers in the family. However, despite the extensive genomic variations identified, our understanding of how these differences contribute to species-specific traits remains limited by the lack of systematic phenotypic and functional data. Many variants occur within coding regions or large-scale rearrangements, potentially impacting gene regulation and expression. Future integrative studies combining transcriptomic analyses, quantitative phenotyping, and functional validation will be essential to clarify genotype–phenotype relationships and the molecular mechanisms driving species diversification in *Anthurium*.

Our genome-wide analyses identified two major genetic groups in *Anthurium*, supported consistently by PCA, phylogenetic, and population structure analyses. However, a moderate pairwise genome-wide *F*_ST_ of 0.17 between these groups indicates partial genetic differentiation with ongoing gene flow. Extensive admixture likely results from repeated hybridization events, while relatively weak artificial selection during breeding has allowed diverse genetic backgrounds to persist. It is important to recognize that hybridization and artificial selection are distinct evolutionary processes: hybridization facilitates genetic mixing, whereas selection reduces diversity at targeted loci. The *π* values of 1.26 × 10^−5^ and 1.98 × 10^−5^ within the two groups further support substantial within-group variation. Hotspot regions identified through π and *F*_ST_ analyses suggest potential targets of selection. Among the 26 *π*-based and 16 *F*_ST_-based candidate genes, most have unknown functions, but four well-characterized genes—*RHF2A*, *WRKY13*, *ARF19*, and *RPE*—highlight key biological processes potentially under selection: *RHF2A* functions in gametogenesis and post-meiotic mitosis, indicating reproductive development as a potential selective target [65]; *WRKY13* [66] and *ARF19* [67] are involved in stem development and hormone-responsive growth regulation, potentially affecting plant structure and development; *RPE*, a key enzyme in the Calvin–Benson cycle, suggests that photosynthetic efficiency may also be subject to adaptive selection [68]. Collectively, these results indicate that selection in *Anthurium* may act on multiple functional pathways, including reproduction, structural growth, and photosynthesis, contributing to both ecological adaptation and important ornamental traits. Similar patterns of weak genetic clustering and pervasive gene flow have been reported in other Araceae species, such as *Colocasia esculent*a and *A. konjac*, where domestication and hybridization shape population structure [69–71]. In contrast, *S. polyrhiza* exhibits stronger genetic divergence linked to ecological specialization, highlighting different evolutionary forces within Araceae [72]. Unlike species exhibiting clearer domestication-driven differentiation, *Anthurium*’s weak population structure likely reflects a combination of low-intensity artificial selection and recurrent interbreeding. These findings underscore the limitations of traditional trait-based horticultural classifications and highlight the necessity of advanced genomic tools to elucidate domestication histories and guide breeding. Although extensive admixture complicates defining discrete genetic groups and may reduce the power of trait association studies, it simultaneously reflects rich genetic diversity that is valuable for breeding and conservation efforts.

The MADS-box gene family, which is critical for floral development [72, 74], includes key regulators, such as *SOC1* [75, 76] and *AGL6* [77]. Comparative analysis of ABC(D)E-model genes across 14 Araceae species revealed lineage-specific loss patterns linked to adaptive innovation. The loss of *SOC1* correlates with primitive spathe evolution, potentially influencing inflorescence architecture and enhancing ecological niche adaptation. Conversely, *AGL6* loss coincided with unisexual flower evolution in class III lineages, suggesting a potential involvement in floral organ differentiation. Notably, the two *Anthurium* species retained both genes, displaying intermediate inflorescence traits (bisexual flowers with developed spathes), indicative of transitional evolution between ancestral and derived states. These findings underscore how gene retention/loss drives morphological diversification: *SOC1* loss enables pollinator-attracting spathe specialization, and *AGL6* loss facilitates reproductive strategy shifts. This highlights genomic flexibility as the cornerstone of floral evolutionary innovation in Araceae.

Spathe coloration in *Anthurium* is influenced by pigment accumulation, including that of anthocyanins, carotenoids, and betalains [78]. Previous studies have indicated that spathe color variation is not dictated by anthocyanins alone [16, 17]. For instance, the cyanidin 3-rutinoside (Cy3R) content and its ratio to pelargonidin 3-rutinoside (Pg3R) correlate with spathe lightness (L*) and the chromatic parameter a* [17]. Consistent with prior findings, we found that the anthocyanin type showed no direct link to color variation in *Anthurium*. However, correlation networks revealed that color parameters (a*, b*, C*) were strongly associated with metabolite ratios, suggesting that spathe coloration arises from coordinated metabolite accumulation rather than from single pigments. While anthocyanins remain the primary contributors to pigmentation [78–80], flavonoids and carotenoids may act as modulators of color intensity and hue. Candidate genes associated with anthocyanin and carotenoid biosynthesis were also identified, highlighting their potential regulatory functions in spathe pigmentation. However, it is important to note that these associations are correlative and do not necessarily indicate causation. Further functional validation—such as gene editing or metabolic profiling—is needed to confirm the direct roles of these metabolites and genes in spathe coloration. This integrative analysis underscores the complex genetic and metabolic framework underlying floral pigmentation in *Anthurium*.

The plant cuticle, which comprises cutin and wax, acts as a critical barrier against environmental stress and water loss [81]. In *Anthurium*, cuticular wax content correlates with vase life. Chemical analysis revealed that spathe waxes primarily consisted of alkanes, fatty acids, and their derivatives, with significant compositional differences between the cultivars during development. Specific alkanes and alkanols can significantly influence wax formation. Cuticular wax biosynthesis involves the elongation of C16/C18 acyl-CoAs to very-long-chain fatty acids via enzymes, such as β-ketoacyl-CoA synthase, reductase, dehydratase, and enoyl-CoA reductase, followed by alkane- or alcohol-forming pathways [82, 83]. In the present study, we found that *CER3* and *KCS1* showed similar expression levels in ‘Alabama’ and ‘Xavia’. The divergent *KCS3* expression in ‘Alabama’ and ‘Xavia’ suggests its regulatory role in wax variation. However, the intricate regulation of wax biosynthesis warrants further investigation to validate these findings [81, 84, 85].

Although key candidate genes such as *CER3*, *KCS1*, *SOC1*, and *AGL6* were identified in relation to cuticular wax formation and floral development, their functional roles were not experimentally validated, which represents a limitation of the current study. Due to the lack of an efficient and stable genetic transformation system in *Anthurium*, gene knockout, overexpression, or other functional assays could not be conducted. Future studies should prioritize the development of suitable genetic tools for *Anthurium* to enable detailed functional characterization of these genes. Alternative approaches, such as virus-induced gene silencing (VIGS), CRISPR/Cas-based gene editing, or heterologous expression systems, may also offer viable strategies for functional validation. Confirming the roles of these candidate genes will be essential for advancing our understanding of the molecular mechanisms underlying spathe wax deposition and floral development in *Anthurium*.

## Conclusion

In conclusion, we reported two high-quality chromosome-level genomes of *Anthurium*, revealing extensive chromosomal rearrangements and species-specific transposon expansions. Population analyses of the 179 accessions identified admixed genetic clusters that were inconsistent with the horticultural groups. Integrated transcriptomic and metabolomic investigations further underscored the critical roles of anthocyanins and alkanes as central determinants of floral traits. Notably, we identified *CER3*, *KCS1*, and *KCS3* as the key regulators of wax biosynthesis. In particular, *SOC1* and *AGL6* homologs have played pivotal roles in the evolutionary diversification of flowers within the Araceae family. These findings revealed the genomic, metabolic, and regulatory bases of *Anthurium* ornamental traits.

## Materials and methods

### Genome assembly and annotation

The *A. andraeanum* ‘Alabama’ (*A. andraeanum*) and *A. scherzerianum* ‘Red Lantern’ (*A. scherzerianum*) for *de novo* assembly were collected from the Chinese Academy of Tropical Agricultural Sciences (Hainan, China) and prepared for sequencing (Supplementary Note 1). All sequencing and associated technical support for genome analysis were provided by Kindstar Sequenon Co., Ltd. Long-read libraries for both *A. andraeanum* and *A. scherzerianum* were prepared and sequenced on the PacBio Revio platform, generating ~96.62 Gb and 92.89 Gb of clean data, respectively. A total of 211.82 Gb of short-read data were generated for *A. andraeanum* using the Illumina HiSeq 4000 platform, while ONT long-read sequencing produced 385.81 Gb of data. For chromosome conformation capture sequencing (Hi-C), libraries were constructed following standard protocols and sequenced on the Illumina NovaSeq 6000 platform (*A. andraeanum*) and BGI MGISEQ-2000 platform (*A. scherzerianum*), producing 379.23 Gb and 484.48 Gb of Hi-C data, respectively. Basic statistics and quality metrics of all sequencing datasets, including sequencing depth, N50 values, and quality scores, are summarized in Tables S1–S3.

Clean paired-end short and HiFi reads were used to estimate the genome size, heterozygosity, and repeat content of the *A. andraeanum* genome using Jellyfish (v2.3.0) [86] and findGSE [87] software. The genome of *A. scherzerianum* was assessed using the same process based on HiFi reads. The genome assembly integrated a combination method based on HiFi reads, Hi-C, and genetic mapping (Fig. S3 and Supplementary Notes 2 and 3).

Genome completeness was estimated using BUSCO (v5.5.0) [88] in the single-copy genes embryophyte_odb10 database. Genome accuracy was evaluated by mapping clean paired-end short and clean HiFi reads to the genome using BWA (v0.7.13-r1126) [89] and Minimap2 (v2.26-r1175) [90], respectively. Genome continuity was assessed by calculating contig N50 lengths and LTR assembly index (LAI) values.

Genome annotation included repeat sequence annotation, protein-coding gene structure and function annotation, and non-coding RNA prediction. More details on genome annotation are provided in Supplementary Notes 4–6.

### Structural variation identification

Genome-wide comparisons were performed between *A. andraeanum* and *A. scherzerianum* using MUMmer4 (v4.0.0rc1) [91]. A delta filter was used to filter the alignment results, and show-snps was used to obtain SNP and InDel information. In addition, structural variations were identified using SyRI (v1.6.3) [92], according to the alignment results of MUMmer4.

### Comparative genomic analysis

A phylogenetic tree was constructed based on the longest protein-coding sequences of *A. andraeanum*, *A. scherzerianum*, and 14 other species (see Supplementary Note 7). The detailed steps for phylogenetic tree construction are described in Supplementary Note 7. Divergence times were estimated using a molecular clock approach calibrated with seven fossil calibration points obtained from the TimeTree database (http://www.timetree.org/). The calibration points included divergence time ranges between *A. thaliana* and *A. trichopoda* (179.9–205.0 Mya), *A. thaliana* and *O. sativa* (142.1–163.5 Mya), *O. sativa* and *Z. marina* (125.3–137.9 Mya), *Z. marina* and *S. polyrhiza* (121.9–135.0 Mya), *P. stratiotes* and *S. polyrhiza* (82.0–117.0 Mya), *Pueraria pedatisecta* and *C. esculenta* (17.0–41.1 Mya), and *L. minor* and *W. australiana* (30.0–55.0 Mya).

The WGD events in *A. andraeanum* and *A. scherzerianum* were identified using a syntenic analysis that reflects the syntenic depth of intra- and interspecific synteny blocks and synonymous substitutions per synonymous site (Ks) analysis. Further details of the WGD analysis are provided in Supplementary Note 8.

The evolutionary history of karyotypes among Araceae species was based on *n* = 6 AMK from Shi *et al.* [55]. WGDI (v0.6.5) [54] was used to complete karyotype mapping between different species with AMK and to infer an evolutionary scenario considering the fewest number of genomic rearrangements (including fusions and fissions) that may have occurred between the AMK and modern Araceae genomes.

### Population genetics analysis

One hundred seventy-nine *Anthurium* germplasm accessions used in this study including 55 form China, 10 from America, 111 from Netherlands, 3 from South America, and 7 from Australia (Table S12). Genomic DNA extracted from 179 *Anthurium* germplasm accessions was subjected to SLAF-seq library construction and high-throughput sequencing. The clean reads were aligned to the *A. andraeanum* genome using BWA (v0.7.13-r1126) [89], and the bam files were sorted and indexed using SAMtools (v1.17) [93]. Duplicate reads were marked using the MarkDuplicates function in the Picard (v2.25.7) package. The GATK (v4.5.0.0) [94] and SAMtools (v1.17) [93] packages were used for SNP calling, and the intersection of the SNPs identified using both methods was considered the final reliable dataset. Subsequently, we filtered SNPs with a missing rate ≤ 50% and MAF > 0.05 [95]. The remaining high-quality SNPs were screened for further analysis.

To construct the phylogenetic tree, we screened a subset of 1,055 4DTv sites in 179 individuals from the entire high-quality SNP dataset (14,006,947 SNPs). Using these SNPs, a maximum likelihood phylogenetic tree was constructed with IQ-TREE 2 (v2.2.2.6). The best-fit substitution model was automatically selected by ModelFinder, and node support was assessed with 1,000 ultrafast bootstrap replicates. We also conducted PCA with 4DTv sites using PLINK (v1.90b7.2) [96]. The population structure was inferred using ADMIXTURE (v1.3.0) [97] with the number of clusters (K) ranging from 2 to 6 and visualized using TBtools (v2.142) [98].

### Transcriptome and co-expression analyses

A total of 66 RNA-seq datasets were obtained, including those for six *A. andraeanum* spathe coloration stages and spadix development stages, and eight horticultural varieties spathe. RNA-seq data were generated from three biological replicates with high repeatability as determined by sample correlation analysis. Clean reads of the spathes of eight horticultural varieties and six developmental stages (S1–S6) of the spathes and spadices of *A. andraeanum* were obtained using fastp (v0.23.4) [99] and aligned to the *A. andraeanum* reference genome using HISAT2 (v2.2.1) [100]. StringTie (v2.2.1) [101, 102] was employed to calculate gene abundance and was normalized to fragments per kilobase of transcript sequence per million base pairs sequenced. DEGs were identified using DESeq2 (v1.42.1) [103] based on gene counts generated using featureCounts (v2.0.6) [104] with a threshold of fold change (FC) ≥ 2 or ≤ 0.5 and a false discovery rate ≤ 0.01. The TO-GCN analysis was employed to investigate the regulatory network related to transcription factors involved in spathe coloration and spadix development, following the method described by Chang *et al*. [105]. The generation process was detailed in Supplementary Note 9.

### Gene family identification

To further study the formation of the unique flower structure of the spadix, MADS-box genes in 14 Araceae species (*S. polyrhiza*, *W. australiana, Lemna minor*, *L. gibba*, *L. japonica*, *L. turionifera*, *C. crispatula*, *A. konjac*, *P. pedatisecia*, *C. esculenta*, *P. stratiotes*, *Z. elliottiana*, *A. andraeanum*, and *A. scherzerianum*) and relative species *Z. marina* were identified. *Arabidopsis* and *O. sativa* MADS-box protein sequences were downloaded from The Arabidopsis Information Resource (TAIR) (https://www.arabidopsis.org/), and Phytozome (https://phytozome-next.jgi.doe.gov/) was used for a BLASTP (v2.14.1+) search against 14 Araceae species and *Z. marina*, with an e-value cutoff of 1e−5. In parallel, MADS (PF00319) and K domain files (PF01486) were downloaded from the Pfam database (http://pfam-legacy.xfam.org/) [106], and HMMER (v3.3) [107] was used to retrieve the protein sequences database of 14 Araceae species and *Z. marina*, using an e-value cutoff of 1e−5 to ensure high-confidence matches. A total of 955 sequences were identified (Table S15) by merging the BLASTP (v2.14.1+) and HMMER (v3.3) results [107]. The protein sequences of the 955 candidate MADS-box genes were analyzed to confirm the conserved structural domains using CDD (https://www.ncbi.nlm.nih.gov/) and SMART (https://smart.embl.de/). Of these, 272 sequences had a complete MADS-box and a K-box, 374 had a complete MADS-box, and 19 had only one K-box (Table S15). Only sequences with a complete MADS-box and K-box were retained for further analysis.

All MADS-box protein sequences were aligned using MAFFT (v7.520) [108], and poorly aligned regions were trimmed using trimAl (v1.4.rev15) [109]. Subsequently, phylogenetic analysis was performed using IQ-TREE2 (v2.2.6) [110]. Type I and II (MIKC-type) MADS-box proteins were distinguished based on the phylogenetic tree (Table S15). The classification of Type II MADS-box genes was carried out following established approaches from previous studies [111–114]. MADS-box protein sequences from *O. sativa* and *Arabidopsis* were used as references. Multiple sequence alignment was performed with MAFFT (v7.520), and poorly aligned regions were removed using trimAl (v1.4.rev15). A maximum likelihood (ML) phylogenetic tree was then inferred with IQ-TREE (v2.2.2.6). Based on the tree topology and the established subgroup classifications in *O. sativa* and *Arabidopsis*, the MADS-box genes in Araceae were assigned to 13 subgroups.

### Determination of color-related traits in *Anthurium* spathes

Analyses of spathe color, anthocyanins, and total flavonoids were conducted. Spathe samples from different developmental stages and *A. andraeanum* varieties were used to determine of anthocyanins and total flavonoids. The spathe samples were ground into a fine powder and extracted using anthocyanin (Yuanye Bio-Technology, Shanghai, China) and flavonoid (Keming Biotechnology, Suzhou, China) detection kits, according to the manufacturer's instructions. Briefly, 0.25 g of powder samples were used for anthocyanin extraction with 8 ml extraction buffer. After extraction at 4 °C in the dark for 20 minutes, the supernatant, collected following centrifugation at 8,000 rpm for 3 minutes, was subjected to spectrophotometry at a wavelength of 530 nm. For flavonoid determination, 0.02 g of the ground sample was extracted using 2 ml of 60% ethanol at 60 °C, shaken for 2 hours, and centrifuged at 14,000 rpm for 10 minutes. Thereafter, the absorbance of the supernatant was measured following the instructions of the flavonoid detection kit, and the absorbances at 510 nm were recorded to obtain the total flavonoid content in the spathe.

Spathe color was quantitatively examined using a colorimeter (NF333; Nippon Denshoku Industries Co. Ltd., Tokyo, Japan) based on the CIELAB color system(L*a*b*), where L* represents perceptual lightness, and a* and b* represent the magnitude of the color shift from green to red and blue to yellow on the surface of an object, respectively. We calculated *C* and *D* using the formula: *C*^2^ = (a*) [27] + (b*) [27] and *D*^2^ = (a*) [27] + (b*) [27] + (L*) [27], respectively.

### Metabolomic analysis of *Anthurium* spathes

For non-targeted metabolomic analysis of spathe samples, fresh spathe samples at the S6 stage from eight horticultural varieties were collected and immediately frozen in liquid nitrogen. The extracts were pre-treated and examined using a UPLC-ESI-MS/MS system (UPLC coupled with 4500 QTRAP-MS; AB Sciex LLC, Framingham, MA, USA), with the setup described in Supplementary Note 8. The relative content of the identified metabolites was calculated as the ratio of the peak area of each metabolite to that of the internal standard and sample weight.

The cuticular wax profiles on the spathe surfaces of ‘Alabama’ and ‘Xavia’ at different developmental stages (S1–S6) were analyzed using a previously described method [84]. The extracted compounds were derivatized and analyzed using gas chromatography–mass spectrometry (7890–5977; Agilent Technologies, Santa Clara, CA, USA). Metabolite annotation was performed based on a spectral search of the National Institute of Standards and Technology (NIST) retention index library. The levels of annotated metabolites are shown as peak areas relative to the internal standard (Table S18).

Carotenoid content was determined in the spathe samples of the eight cultivars using MetWare (http://www.metware.cn/), based on the AB SCIEX QTRAP 6500 LC–MS/MS platform. The spathe samples were extracted and prepared for LC–MS/MS analysis as described in the Supplementary Note 10.

### Cuticle staining analyses of spathes at different developmental stages

Frozen section analysis was performed to observe the cuticle structure of the spathe. The middle sections of the fresh spathe of ‘Alabama’ and ‘Xavia’ at S1–S6 were collected and fixed in formaldehyde-acetic acid-alcohol solution. Thereafter, sections were dried using filter paper and frozen in liquid nitrogen. Next, the sections were soaked in OCT embedding agent on a freezing table and sliced to 10-μm thickness using a cryostat (CRYOSTAR NX50; Epredia, Portsmouth, NH, USA). The slices were warmed and rinsed before staining with oil-saturated O liquid. Subsequently, the stained slices were sealed and observed under a microscope (NIKON ECLIPSE E100; Nikon, Tokyo, Japan).

## Supplementary Material

Web_Material_uhaf316
