# Supplementary material for: Integrative multi-omics analysis reveals the genetic architecture of floral traits in Anthurium
Source: Hortic Res. 2025 Nov 8;13(1):uhaf316. doi: 10.1093/hr/uhaf316 (PMC12871434; doi:10.1093/hr/uhaf316)
Supplement: Web_Material_uhaf316 [file web_material_uhaf316.zip › Supplementary information and figure_v16_clean.docx]

**SUPPLEMENTAL INFORMATION**

1. **Supplementary materials and methods**

## **Supplementary Note 1: Plant materials, DNA and total RNA extraction**

The genomic DNA from fresh young leaves were extracted using cetyltrimethylammonium bromide (CTAB) protocolz and quantified using NanoDrop and Qubit instruments. Five organs (leaf, root, stem, spadix and spathe) from *Anthurium andraeanum* and *A. scherzerianum* were sampled for RNA sequencing (RNA-seq) to assist genome annotation. In order to explore the coloration of spathe, bract samples were collected from eight *A. andraeanum* horticultural varieties of spathe with different color including ‘Acropolis’ (white), ‘Alabama’ (red), ‘Pink Champion’ (pink), ‘Sierra’ (orange), ‘Purple Princess’ (purple), ‘Choco’ (brown), ‘Black Queen’ (black), and ‘Midori’ (green), were collected for metabolomic analysis. And six spathe developmental stages of *A. andraeanum* ‘Alabama’ and *A. andraeanum* ‘Xavia’ were collected: stage 1 (S1) with a pedicel length of 1–2 cm, stage 2 (S2) with a pedicel length of 2–4 cm, stage 3 (S3) with a pedicel length of 7–9 cm, stage 4 (S4) with a pedicel length of 12–15 cm, stage 5 (S5) when the spathe is slightly curled and beginning to unfold, and stage 6 (S6) when the spathe is fully open. The bract corresponding to all six stages of both cultivated varieties were used for cuticular wax analysis using for SPME-GC-MS, while the bract and spadix from *A. andraeanum*, were subjected to transcriptome sequencing. All samples were harvested and immediately frozen in liquid nitrogen and then preserved at −80℃for further usage.

## **Supplementary Note 2: Genome assembly**

The clean HiFi reads of *A. andraeanum* and *A. scherzerianum* were initially assembled into contig-level draft genome using Hifiasm (v0.19.5-r592) software. Subsequently, the contig-level assemblies were removed contigs contaminated with bacterial and plant plastids were removed by performing BLASTN (v2.14.1+) against the bacterial and plant plastid sequence libraries from the NCBI RefSeq database. Next, haplotyped duplication sequences were filtered using purge_dups (v1.2.5) to obtain haplotype removal assemblies. The clean Hi-C data were aligned to the haplotype removal assemblies by Juicer (v1.6). Then those uniquely mapped and valid paired-end reads were assembled by 3D-DNA pipeline (201008). Juicebox (v1.11.08) was used to manually correct the assembly errors to get the chromosome assemblies. The ALLHiC algorithm (v0.9.8) was used to plot the heat map of genome interactions Finally, the clean HiFi reads were utilized to fill gaps by applying quarTeT (v1.1.5) software.

## **Supplementary Note 3: Genetic map construction**

The segregated population consisted of 200 progenies generated from the controlled crossing between *A. andraeanum* cv. Alabama (♀) and Acropolis (♂). The genomic DNA was extracted from the progeny leaves according to the modified CTAB method. The Specific-Locus Amplified Fragment (SLAF) marker identification, genotyping, and genetic map construction were performed following the previous procedures (Sun et al., 2013).

## **Supplementary Note 4: Repeat sequence annotation**

The repeat sequence was annotated with a combination of *de* *novo* and homology-based methods. The *de novo* repeat library of the target genome was constructed using RepeatModeler (v2.0.5) with the parameter “-LTRStruct” (Flynn et al., 2020). Then library was combined with the RepBase database (http://www.girinst.org/repbase) (Bao et al., 2015) for searching homologous repeats using Repeatmasker (v4.1.5) (Chen, 2004). Each genome assembly was hard- and soft-masked by RepeatMasker (v4.1.5).

## **Supplementary Note 5: Protein-coding gene structure and functional annotation**

A strategy combining *ab initio* gene prediction, homology-based gene prediction and transcriptome-based gene prediction was used to predict protein-coding genes. Augustus (v3.5.0) (Hoff and Stanke, 2019), SNAP (v2017-03-01) (Korf, 2004), GALBA (v1.0.8) (Stanke et al., 2006; Buchfink et al., 2015; Hoff and Stanke, 2019; Brůna et al., 2023), and GETA (v2.5.5) were employed for *ab initio* gene prediction. The homology-based gene prediction was performed using Miniport (v0.12-r237) (Li, 2023) and CD-HIT (v4.8.1) (Li and Godzik, 2006; Fu et al., 2012) based on a non-redundant homologous protein library constructed from the model plants or related species, including *Arabidopsis* *thaliana*, *Oryza sativa*, *Solanum lycopersicum*, *Vitis vinifera*, *Nymphaea colorata*, *Elaeis guineensis*, *Acorus gramineus*, *Amorphophallus konjac*, *Colocasia esculenta*, *Lemna gibba*, *L. japonica*, *L. minor*, *L. turionifera*, *Pistia stratiotes*, *Spirodela polyrhiza*, *Wolffia australiana* and *Zantedeschia elliottiana*. For transcriptome-based gene prediction, the RNA-seq reads from five organs (leaf, root, stem, spadix and spathe) were mapped to its genome using HISAT2 (v2.2.1) (Kim et al., 2015) and assembled transcripts by StringTie (v2.2.1) (Pertea et al., 2015; Pertea et al., 2016). Then, TransDecoder (v5.7.1) was used to identify candidate coding regions in transcript sequences. In addition, Trinity (v2.15.1) (Grabherr et al., 2011; Haas et al., 2013) was used to assemble transcripts and followed by using PASA (v2.5.3) (Haas et al., 2003) for gene model prediction. Finally, the non-redundant gene models from prediction were integrated by EVidenceModeler (v2.1.0) (Haas et al., 2008). The protein-coding annotations were assessed by BUSCO. Functionally gene annotation was performed according to the best matches by blasting GO, COG & KOG, SwissProt, TrEMBL, Pfam, Interproscan, eggNOG and NCBI non-redundant protein databases.

## **Supplementary Note 6:** **Non-coding RNA prediction**

For non-coding RNA prediction, the tRNA genes were identified using tRNAscan-SE (Chan et al., 2021), the rRNA sequences were predicted by using RNAmmer (v1.2) (Lagesen et al., 2007), and miRNAs, snoRNAs, and snRNAs were annotated with Infernal (v1.1) (Nawrocki and Eddy, 2013) based on the Rfam (v14.5) database (Griffiths-Jones et al., 2005; Kalvari et al., 2018).

## **Supplementary Note 7: Phylogenetic analysis**

*A. andraeanum*, *A. scherzerianum* and other 14 species were selected to constructed the phylogenetic tree, including *Amborella trichopoda*, *A.* *thaliana*, *O. sativa*, *Phalaenopsis equestris*, *Zostera marina*, *S. polyrhiza*, *W. australiana,* *L. minor*, *Pinellia pedatisecta*, *Cryptocoryne crispatula*, *A. konjac*, *C. esculenta*, *P. stratiotes*, and *Z. elliottiana*. Firstly, the single-copy orthologs were identified by OrthoFinder (v2.5.5) (Emms and Kelly, 2015; Emms and Kelly, 2019). Next, the multiple sequence alignment results generated by OrthoFinder (v2.5.5) (Emms and Kelly, 2015; Emms and Kelly, 2019), filtering out the poorly aligned regions using trimAl (v1.4.rev15) (Capella-Gutiérrez et al., 2009) and then subjected to RAxML(v8.2.13) (Stamatakis, 2014) to generate an evolutionary tree. Divergence times between species were estimated using MCMCtree (v4.10.7) from the PAML (v4.10.7) package with independent rate and GTR + G substitution model (Yang, 2007). The final phylogenetic tree was visualized using FigTree (v1.4.3). Gene family expansion and contraction analyses were conducted using CAFÉ (v5.0.0) (Mendes et al., 2021).

## **Supplementary Note 8: Whole-genome duplication analysis**

For the syntenic analysis, intraspecific and interspecific synteny blocks were defined by MCScan via JCVI (Tang et al., 2024) based on core-orthologous gene sets identified by BLASTP (v2.14.1+). Meanwhile, the syntenic analysis between *A. andraeanum*, *A. scherzerianum*, *A. konjac*, *C. esculenta*, *L. gibba*, *L. japonica*, *L. minor*, *L. turionifera*, *P. stratiotes*, *S. polyrhiza*, *W. Australiana*, *Z. elliottiana*, *P. pedatisecta* and *C. balansae* with a reconstructed ancestral monocot karyotype (AMK) were also performed (Shi et al., 2022). For *Ks* analysis, *Ks* values of all syntenic paralogous/orthologous pairs were calculated using WGDI (v0.6.5) (Sun et al., 2021).

## **Supplementary Note 9: TO-GCN analysis for the spathes and spadices in *A. andraeanum***

Genes were defined as DEGs if they met the criteria of a fold change (FC) ≥ 2 or ≤ 0.5 with a false discovery rate (FDR) ≤ 0.05 between any two of the six developmental stages (S1–S6) of the spathes and spadices of *A*. *andraeanum*, and had a TPM value > 0.5 in at least one sample. Based on these thresholds, 11,643 DEGs in spathes and 10,178 DEGs in spadices were identified and subsequently used for the construction of TO-GCNs. Pearson’s correlation coefficient was used to determine gene connectivity within each GCN. To initiate the TO-GCNs, one ERF transcription factor (AanV1_03G007710) for spathes and one AP1 transcription factor (AanV1_12G008020) for spadices were selected based on their high expression at stage S1 and a progressive decline toward stage S6. A correlation coefficient cutoff of 0.97 was applied for spadices and 0.95 for spathes. Finally, gene lists were generated for each TO-GCN level across six time points, including TF matrices, non-TF gene matrices, the initial node TF, and the applied cutoff values. This process yielded 11 and 10 time-ordered expression levels for spadices and spathes, respectively, reflecting the inferred temporal progression of gene expression throughout the flowering process.

## **Supplementary Note 10: Metabolomic profiling on spathe of different cultivars**

Lyophilized spathes were ground into powder using a grinder (MM 400, Retsch, Haan, Germany) with steel beads at 30 Hz for 1.5 min. 50 mg of tissue powder was extracted in 1.2 ml of pre-cooled 70% methanolic aqueous. The mixture was vortexed for 30 seconds every 30 min for a total of six times and then centrifuged at 12000 rpm for 3 min. The supernatant was filtered through a 0.22 μm microporous membrane and stored in the injection vial for UPLC-MS/MS analysis.

A UPLC-ESI-MS/MS system (UPLC coupled with 4500 QTRAP-MS, AB Sciex LLC, MA, U.S.A) was used for the relative quantification of non-targeted metabolic features in bracts. The metabolite separation was conducted through an SB-C18 column (1.8 µm, 2.1 mm × 100 mm, 40 ℃, 0.35 mL/min, Agilent) with an injection volume of 4 µL. The mobile phase A consisted of water with 0.1% (v/v) formic acid, and phase B contained acetonitrile with 0.1% formic acid. The linear gradient program was set as follows: 0-9 min, 95% -5% A; 9-10 min, 95%; 10-11.1 min, 5%-95% A; 11.1-14.0 min, 95% A. The effluent was connected to the MS system with positive or negative mode. The MRM mode was applied for the identification and quantification of analytes against the NIST2011 and Metare’s metabolite database (MWDB, Metware Biological Science and Technology Co., Ltd. Wuhan, China). The relative contents of identified metabolites were calculated as the ratio of the peak area of each metabolite to that of internal standard and sample weight.

For the cuticular wax analysis, the spathes were cut into a size of 1-4 cm^2^ and frozen in liquid nitrogen. 0.5 g of samples were soaked in an extraction solvent containing methanol and chloroform (1:3, v: v) for wax extraction. After ultrasonic extraction for 2 min at room temperature and centrifuge, the chloroform phase was collected and transferred to a fresh 2 ml tube. Then the chloroform phase was evaporated using a continuous nitrogen gas flow. Compounds were dissolved and derivatized with 50 μl pyridine and 60 μl bis-N, N-(trimethylsilyl) trifluoroacetamide (BSTFA, Sigma, MO, USA) at 70 °C for 40 min. The composition and contents of cuticular wax were analyzed by Gas chromatography-mass spectrometry (7890-XXX, Agilent, USA) with a 30 m × 250 µm × 0.25 µm HP-5 MS capillary column. Helium was used as the carrier gas at 1 ml min^−1^. The oven temperature setup was programmed as follows: Sample injection at 50 °C and holding for 1 min, an increase of 8 °C min^−1^ from 50 to 120°C, an increase of 5 °C min^−1^ from 120 to 300 °C and then holding for 10 min. Metabolite annotation was performed based on spectral searching against NIST retention index library. The levels of annotated metabolites were shown as relative content of peak area to internal standard.

For the determination of carotenoids, spathe samples were freeze-dried and ground into powder using pre-cooled metal beads (30 Hz, 1.5 min). 50 mg powder was weighted and extracted with 0.5 mL mixed solution of n-hexane: acetone: ethanol (1:1:1, v/v/v). The extract was vortexed for 20 min at room temperature. The supernatants were collected after centrifuged at 12000 r/min for 5 min at 4°C. The residue was re-extracted by repeating the above steps again under the same conditions. Then, the supernatant was evaporated to dryness, followed by reconstituting in the mixed solution of MeOH/MTBE (1:1, v/v). The solution was filtered through a 0.22 μm membrane filter and analyzed using an UPLC-APCI-MS/MS system (UPLC， ExionLC™ AD，https://sciex.com.cn/ ; MS，Applied Biosystems 6500 Triple Quadrupole, https://sciex.com.cn/ ). The analytical conditions were as follow, LC: column, YMC C30(3 μm, 100 mm×2.0 mm i.d); solvent system, methanol：acetonitrile (1:3, v/v) with 0.01% BHT and 0.1% formic acid (A), methyl tert-butyl ether with 0.01% BHT (B); gradient program, started at 0% B (0-3 min), increased to 70% B (3-5 min), then increased to 95% B (5-9 min), finally ramped back to 0% B (10-11 min); flow rate, 0.8 mL/min; temperature, 28°C; injection volume: 2 μL. The APCI-MS/MS conditions were setup as follows: Linear ion trap (LIT) and triple quadrupole (QQQ) scans were acquired on a triple quadrupole-linear ion trap mass spectrometer (QTRAP), QTRAP® 6500+ LC-MS/MS System, equipped with an APCI Heated Nebulizer, operating in positive ion mode and controlled by Analyst 1.6.3 software (Sciex). The APCI source operation parameters were as follows: ion source, APCI+; source temperature 350°C; curtain gas (CUR) was set at 25.0 psi. Carotenoids were analyzed using scheduled multiple reaction monitoring (MRM). Data acquisitions were performed using Analyst 1.6.3 software (Sciex). Multiquant 3.0.3 software (Sciex) was used to quantify all metabolites. Mass spectrometer parameters including the declustering potentials (DP) and collision energies (CE) for individual MRM transitions were done with further DP and CE optimization. A specific set of MRM transitions were monitored for each period according to the metabolites eluted within this period.

**Supplemental Figures**

**
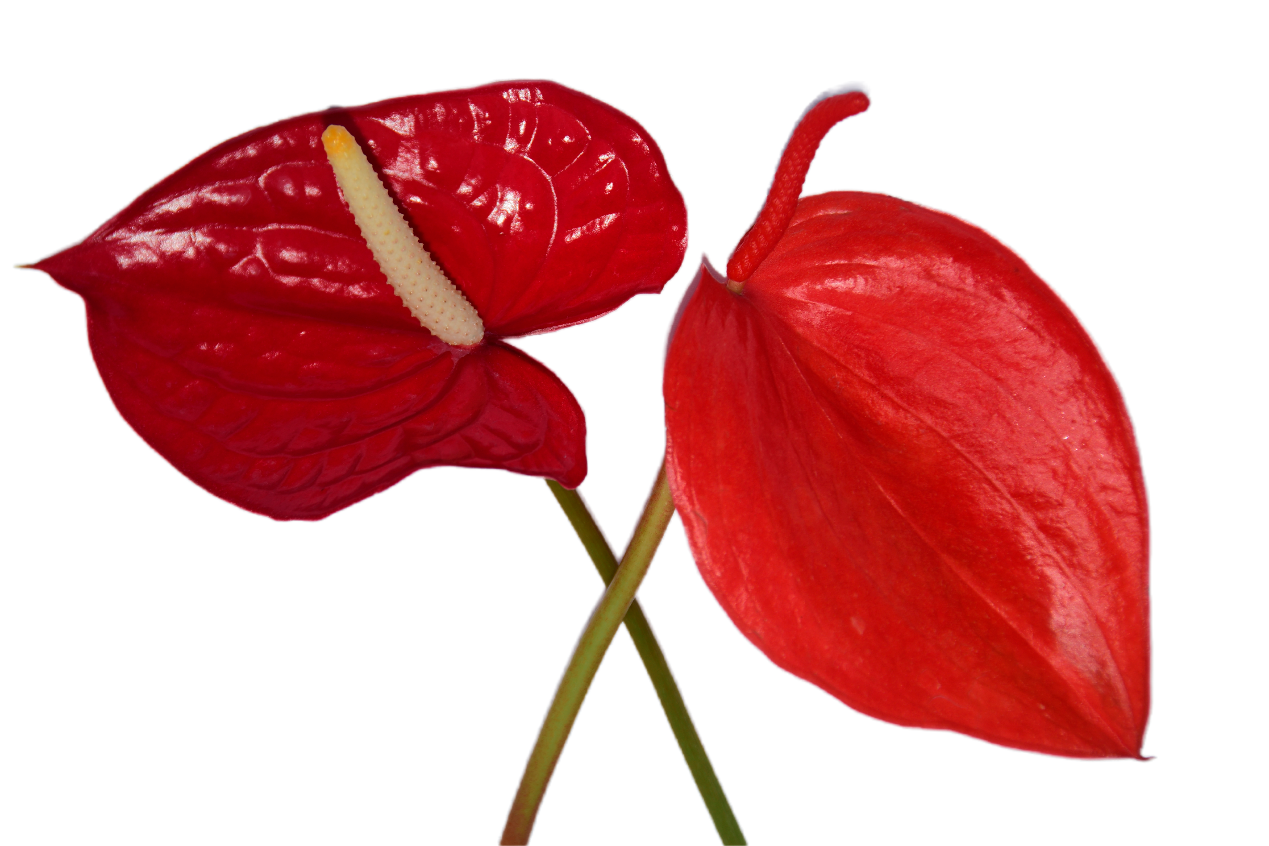
Figure S1. Photographs of *A. andraeanum* ‘Alabama’ (left) and *A. scherzerianum* ‘Red Lantern’ (right).**

**
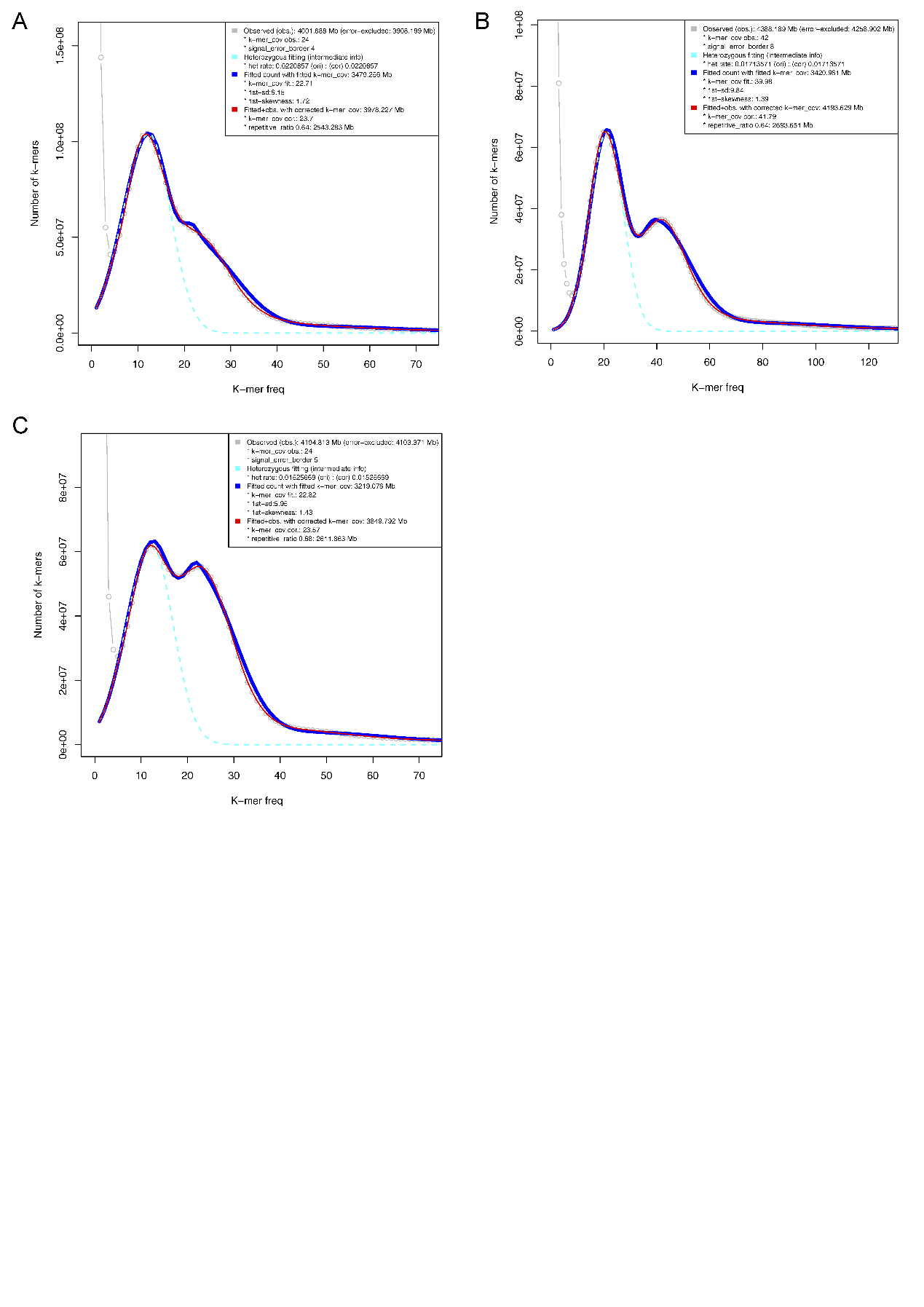
Figure S2. K-mer count distribution and estimation of genome size and heterozygosity of *A. andraeanum* ‘Alabama’ (A and B) and *A. scherzerianum* ‘Red Lantern’ (C).**

**
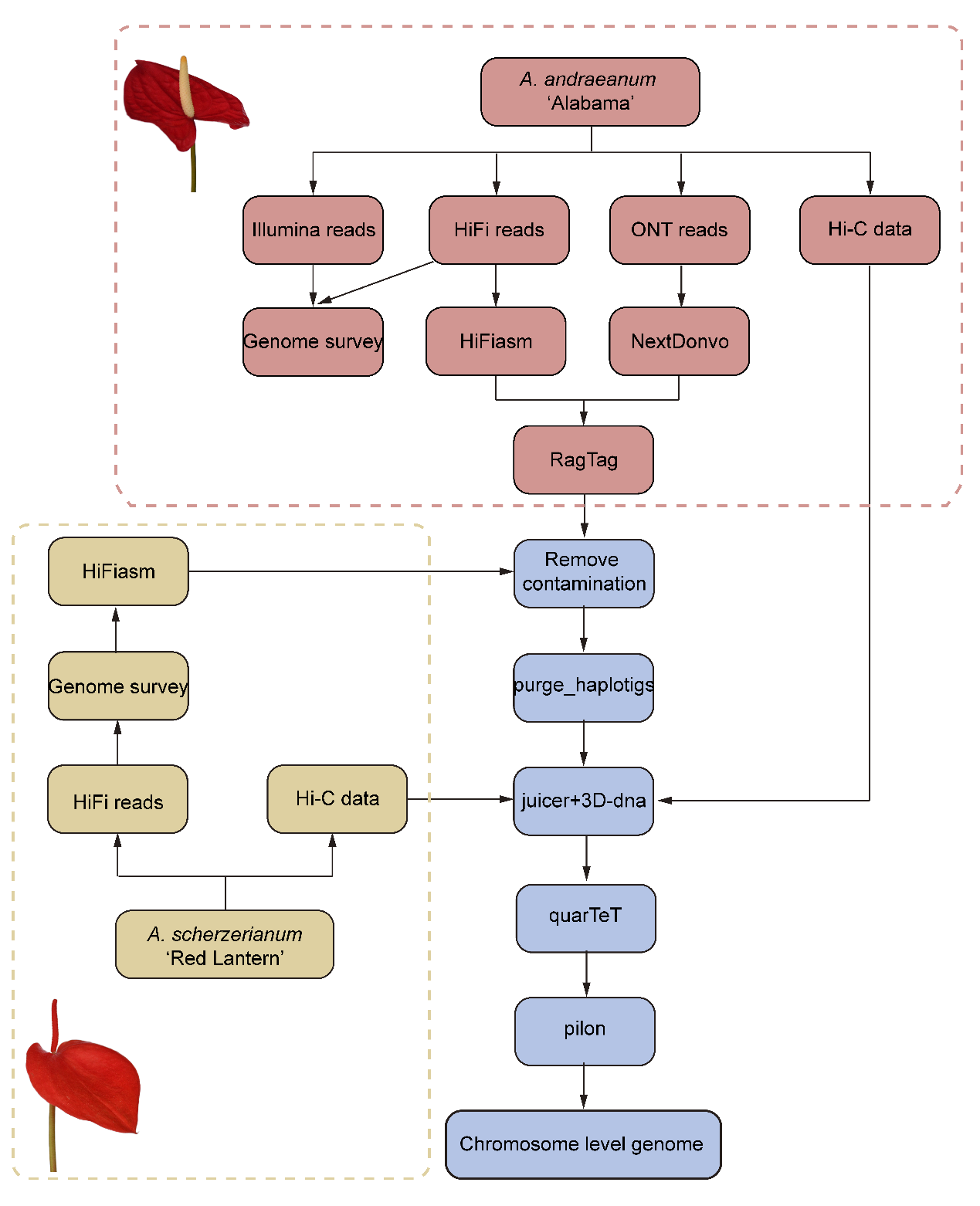
Figure S3.** **Assembly flowchart of two *Anthurium* species.**

**
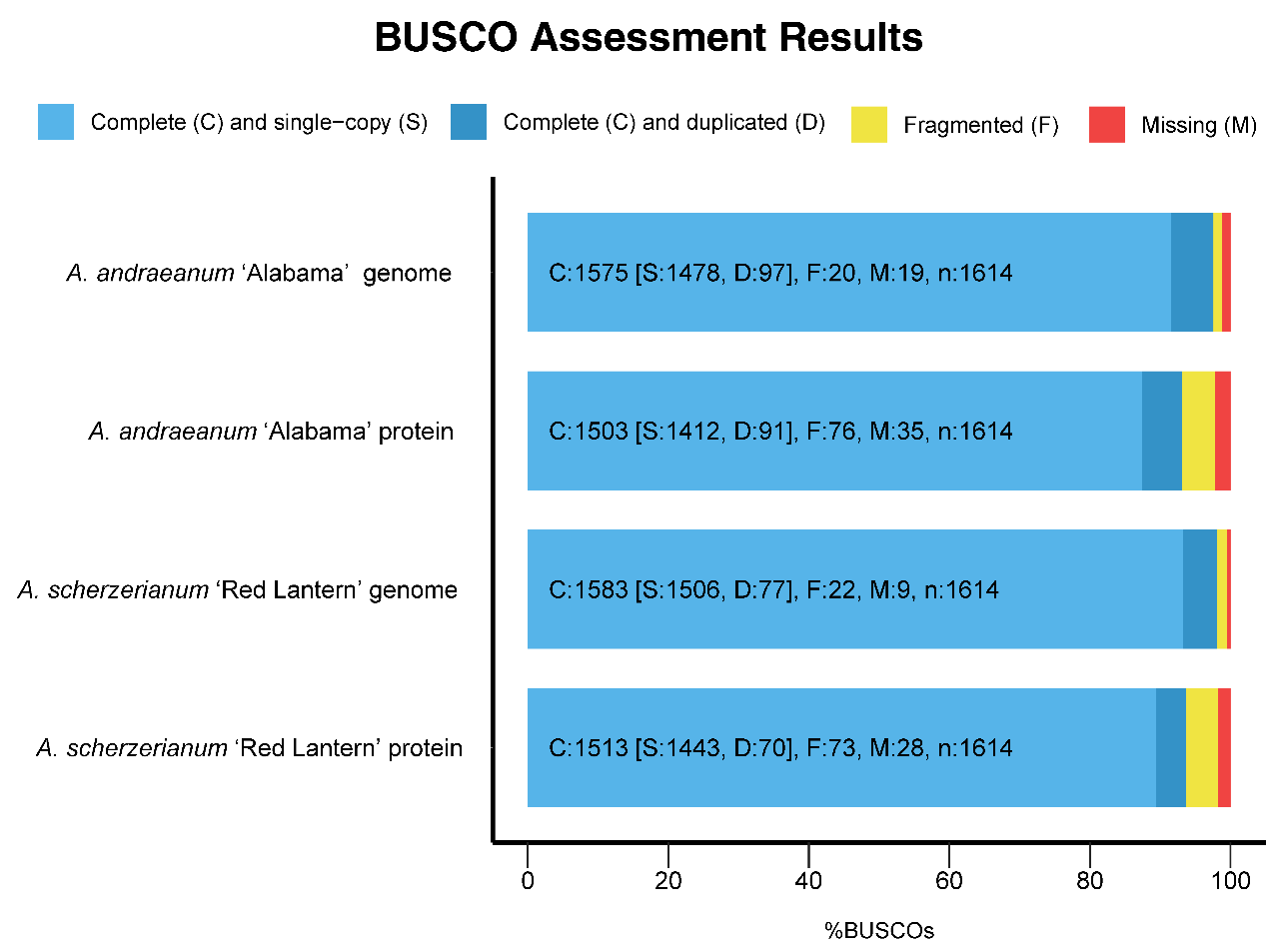
Figure S4. Busco assessment results of genome and predicted protein of two *Anthurium* species.**

**
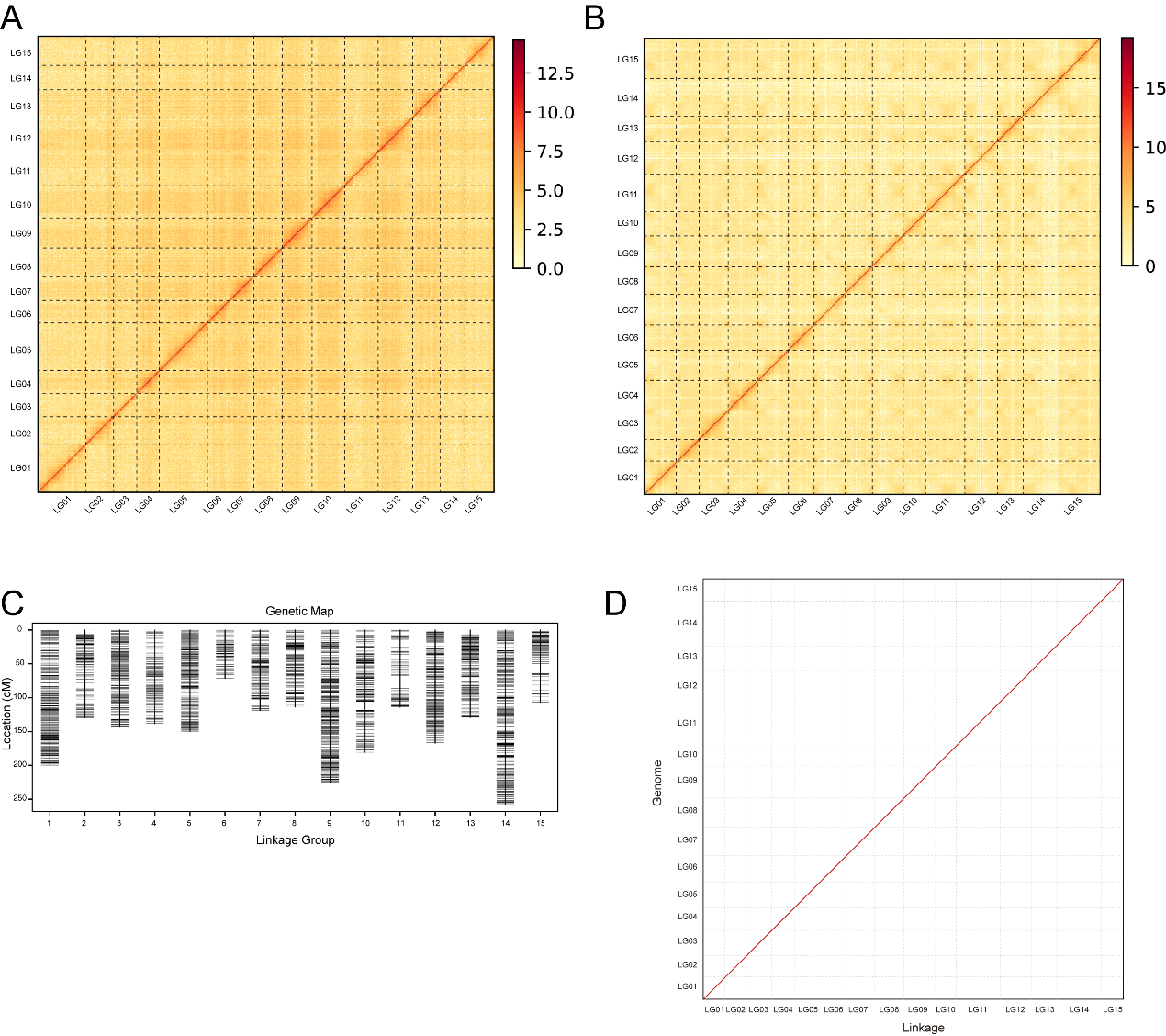
Figure S5. Chromosome-level genome assembly assessment of two *Anthurium* species.** (A) and (B) The plots show genome-wide analyses of chromatin interactions at 800-kb resolution in the genomes of *A. andraeanum* ‘Alabama’ and *A. scherzerianum* ‘Red Lantern’. (C) Genetic map of *A. andraeanum* ‘Alabama’. (D) Collinearity between genetic map and genome of *A. andraeanum* ‘Alabama’.

**
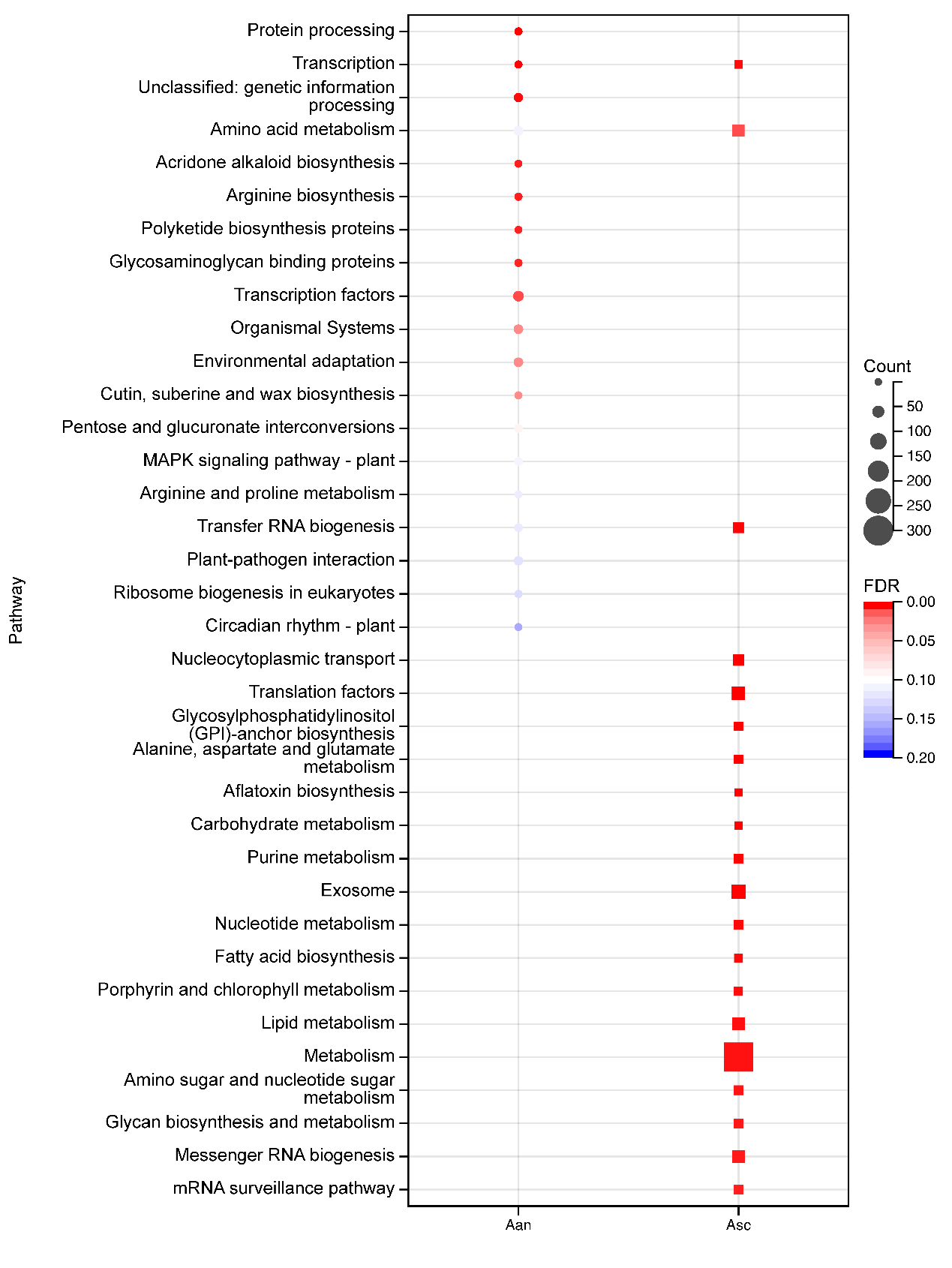
Figure S6. Analysis of unique paralogs enrichment in *A. andraeanum* ‘Alabama’ (Aan) (A) and *A. scherzerianum* ‘Red Lantern’ (Asc) (B) genomes.**

**
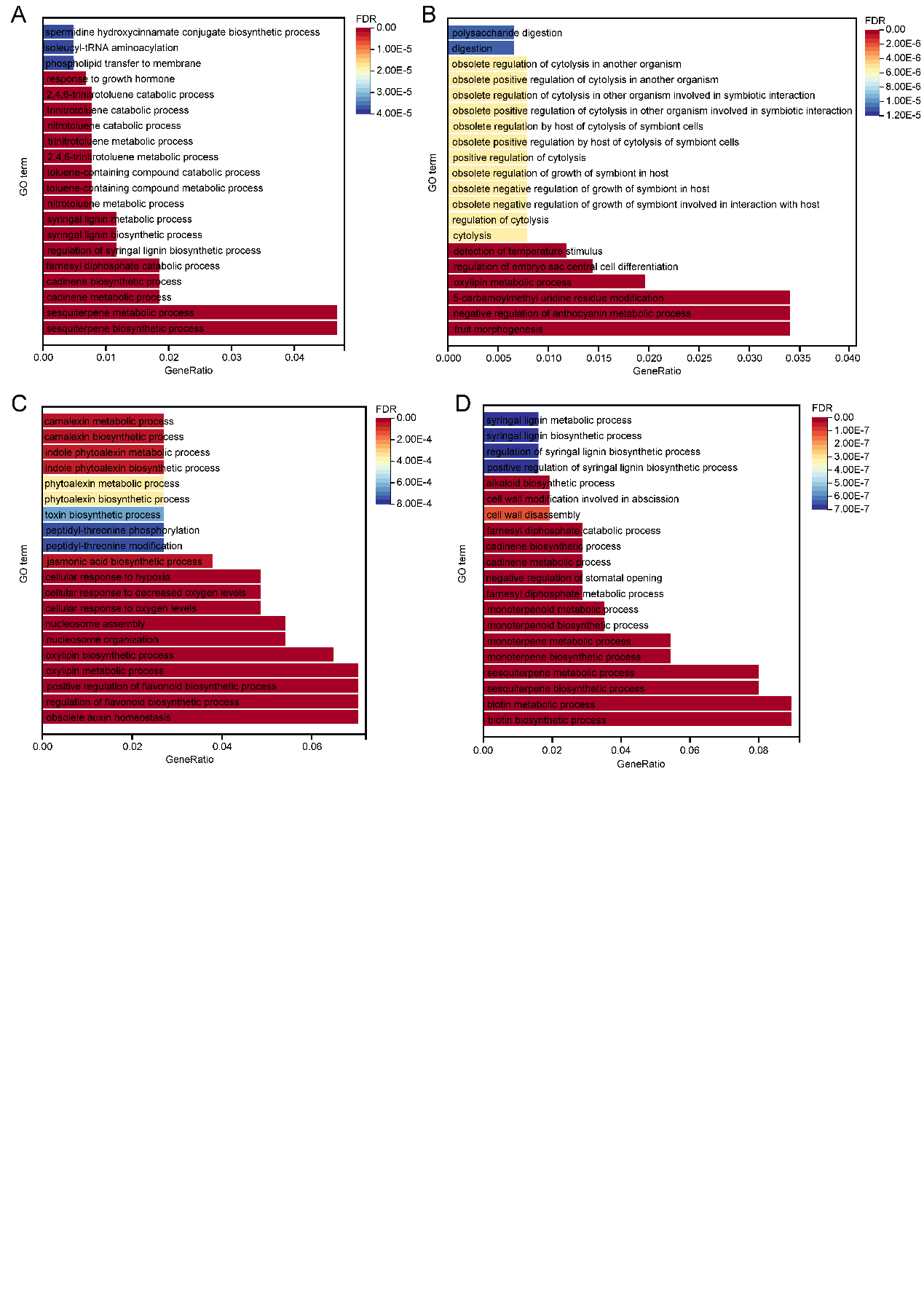
****Figure S7.** **Analysis of expanding and contracting** **gene families enrichment in *Anthurium* genomes.** (A) and (B) Plots of the top 20 enriched GO terms using 1,454 and 1,131 expansion gene families in *A. andraeanum* ‘Alabama’ and *A. scherzerianum* ‘Red Lantern’ genomes are shown. (C) and (D) Plots of the top 20 enriched GO terms using 594 and 611 contraction gene families in *A. andraeanum* ‘Alabama’ and *A. scherzerianum* ‘Red Lantern’ genomes are shown.

**
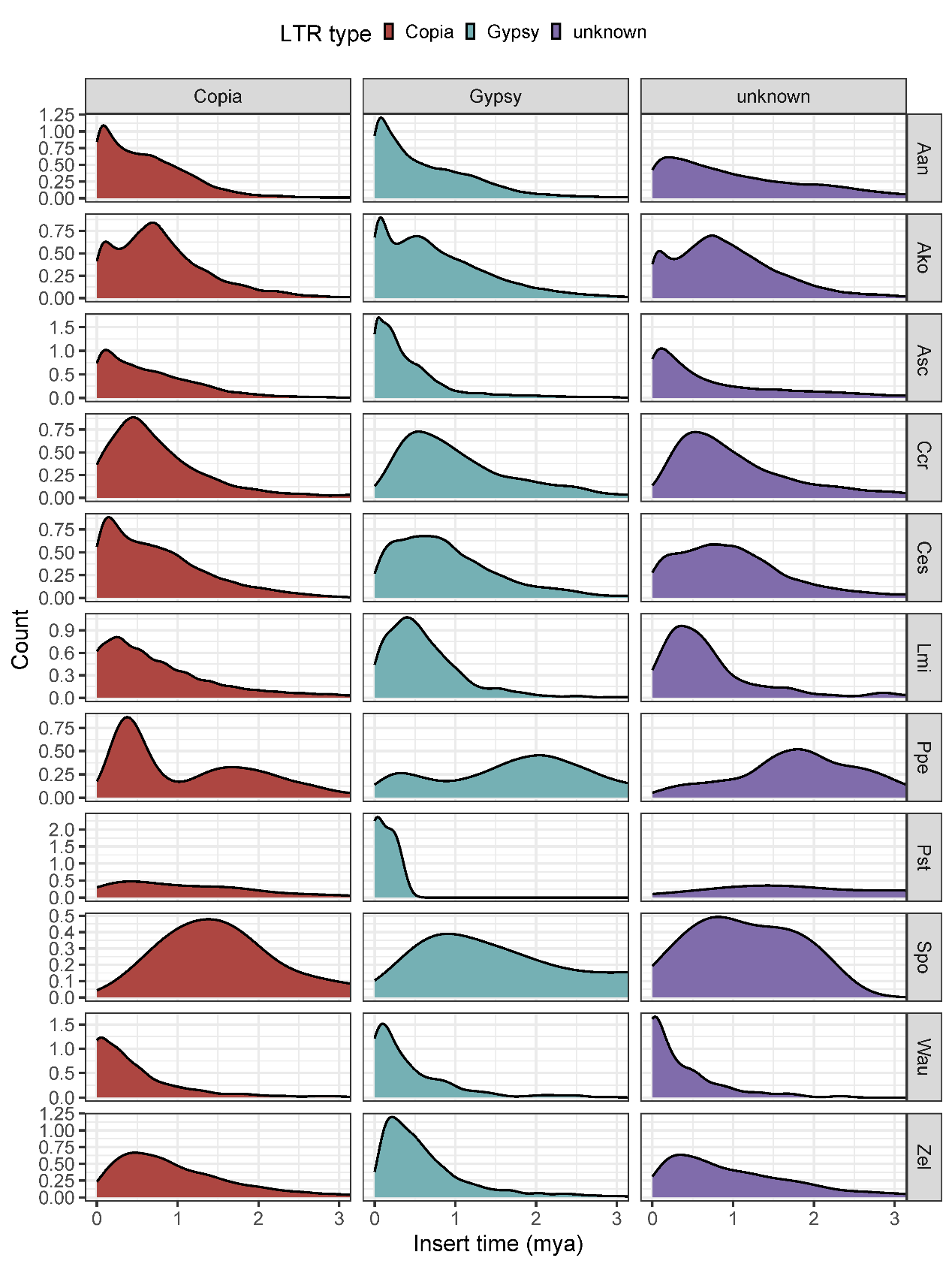
Figure S8.** **The insertion times of LTRs in Araceae species.** From top to bottom: *A. andraeanum* ‘Alabama’ (Aan), *Amorphophallus konjac* (Ako), *A. scherzerianum* ‘Red Lantern’ (Asc), *Cryptocoryne crispatula* (Ccr), *Colocasia esculenta* (Ces), *Lemna minor* (Lmi), *Pinellia pedatisecta* (Ppe), *Pistia stratiotes* (Pst), *Spirodela polyrhiza* (Spo), *Wolffia australiana* (Wau), and *Zantedeschia elliottiana* (Zel) genomes.


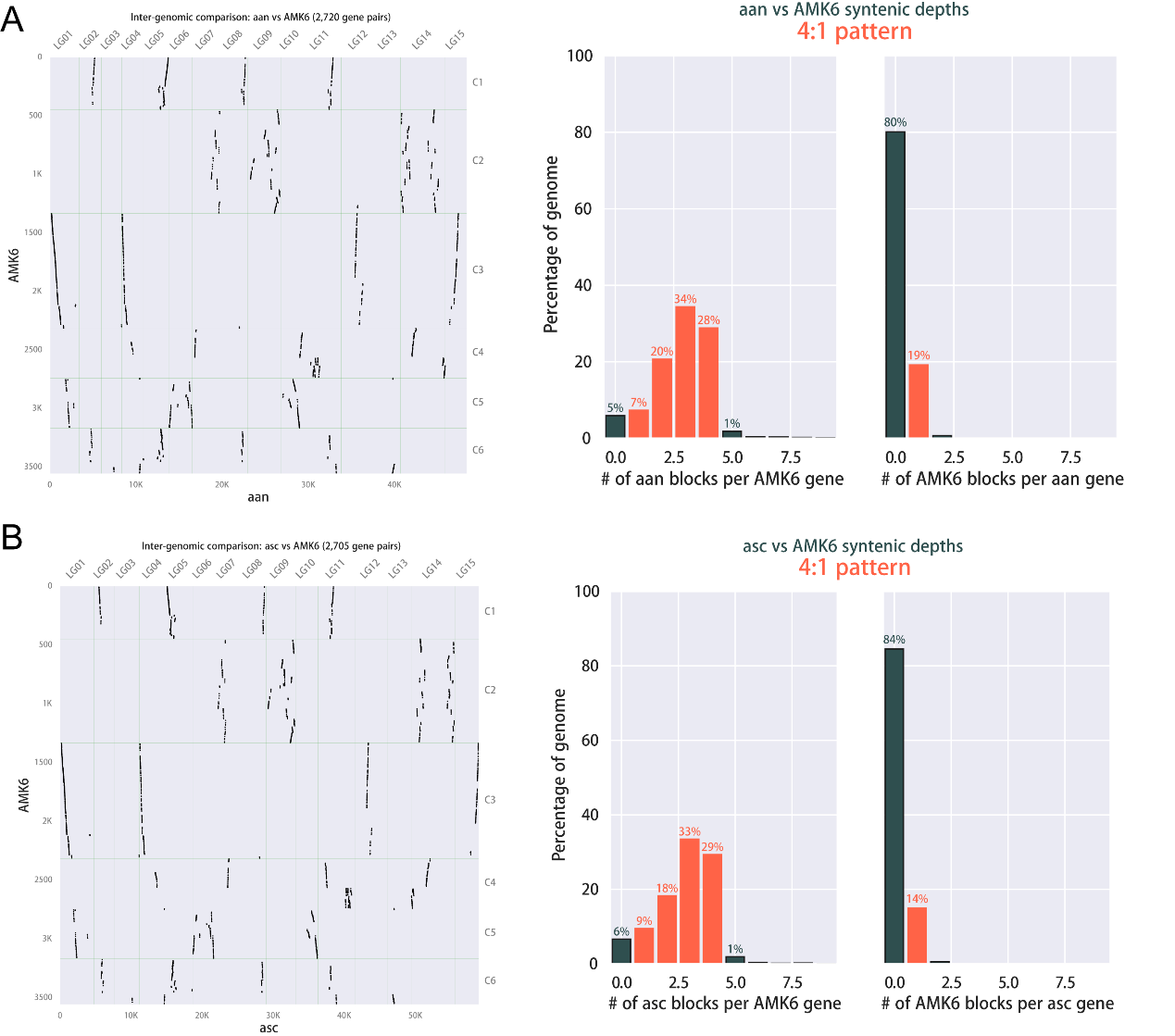


**Figure S9. Syntenic analysis between *Anthurium* genomes and the ancestral monocot karyotype (AMK).** (A) *A. andraeanum* ‘Alabama’ (aan) and (B) *A. scherzerianum* ‘Red Lantern’ (asc) both show a clear 4:1 synteny pattern in the dot plots and corresponding syntenic depth histograms, supporting two rounds of whole-genome duplication.


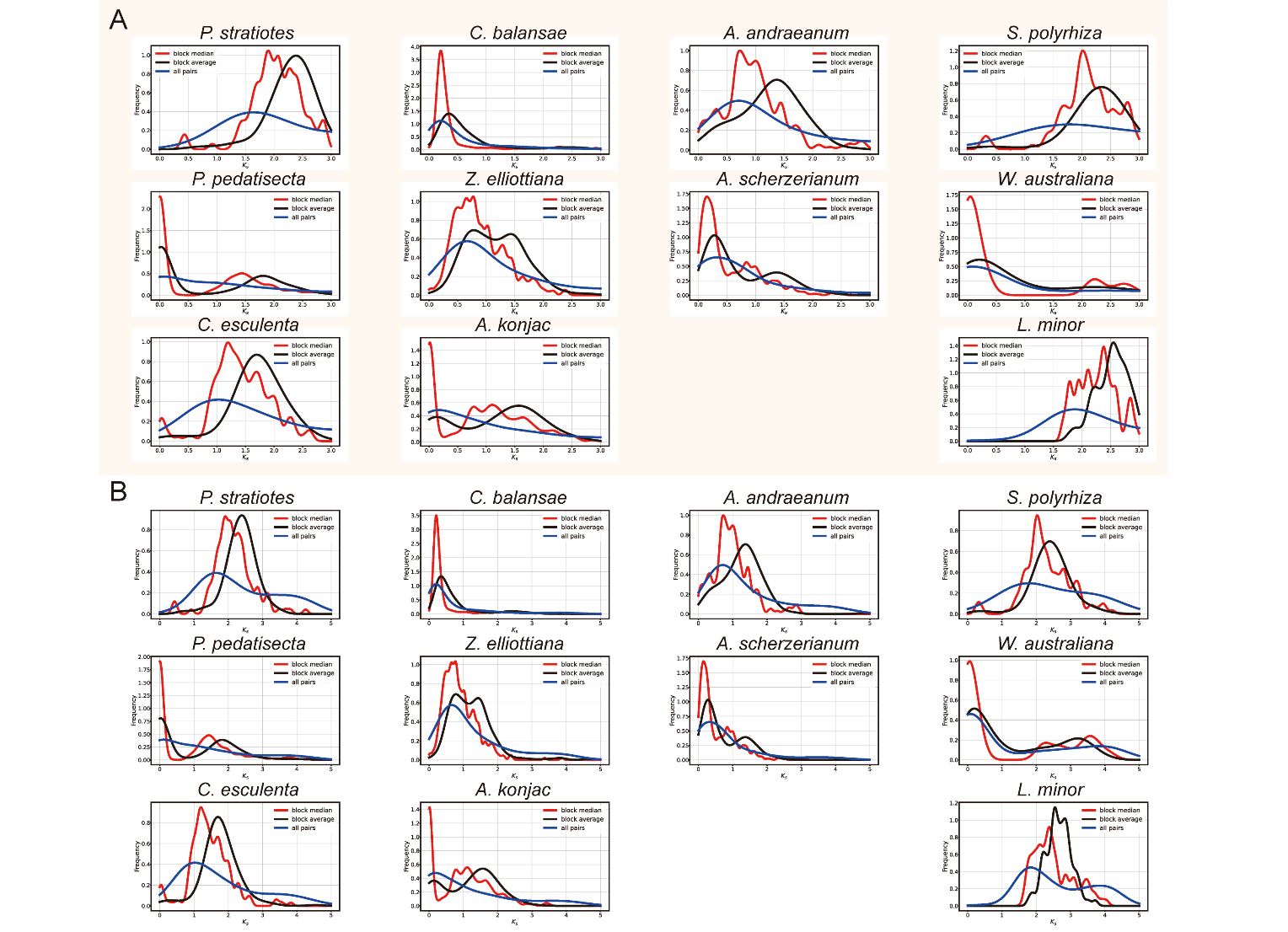
**Figure S10. Ks distribution profiles of 11 Araceae species.** Peaks indicate ancient whole-genome duplication (WGD) events within the family. (A) Ks range: 0–3. (B) Ks range: 0–5.

**
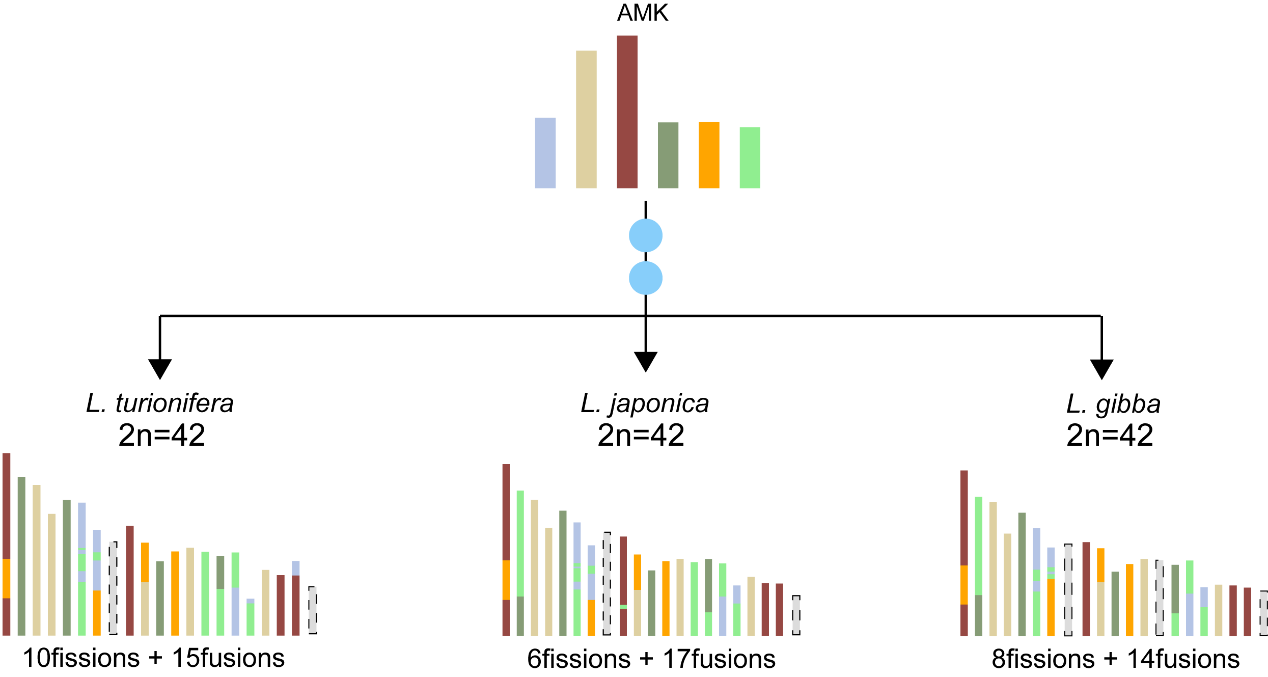
Figure S11. Evolutionary scenario of three *Lemna* genomes based on AMK.** Comparative karyotype analysis indicates that the *Lemna* genomes have maintained a relatively stable karyotype over evolutionary time. Chromosomes shown with dashed lines represent regions not covered by AMK. The two blue circles indicate two WGD events.

**
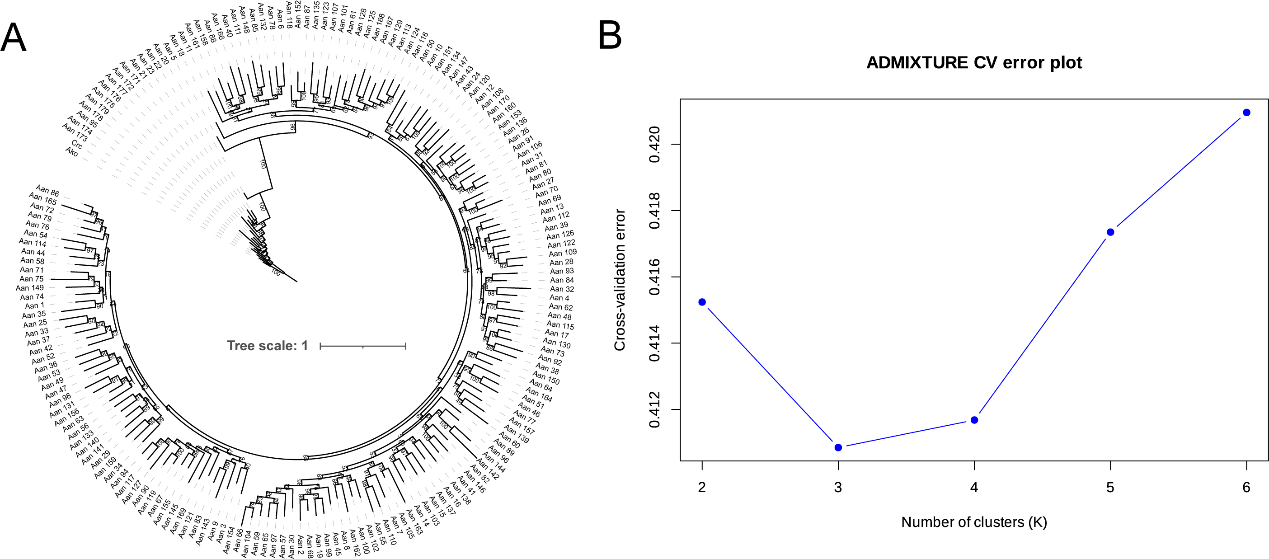
Figure S12. Population genetic analyses based on high-quality SNPs.** (A) Maximum likelihood phylogeny with 1,000 bootstrap replicates showing two main clades. Bootstrap values from 1,000 replicates are shown at each node, indicating the level of branch support. (B) Cross-validation (CV) error plot.

**
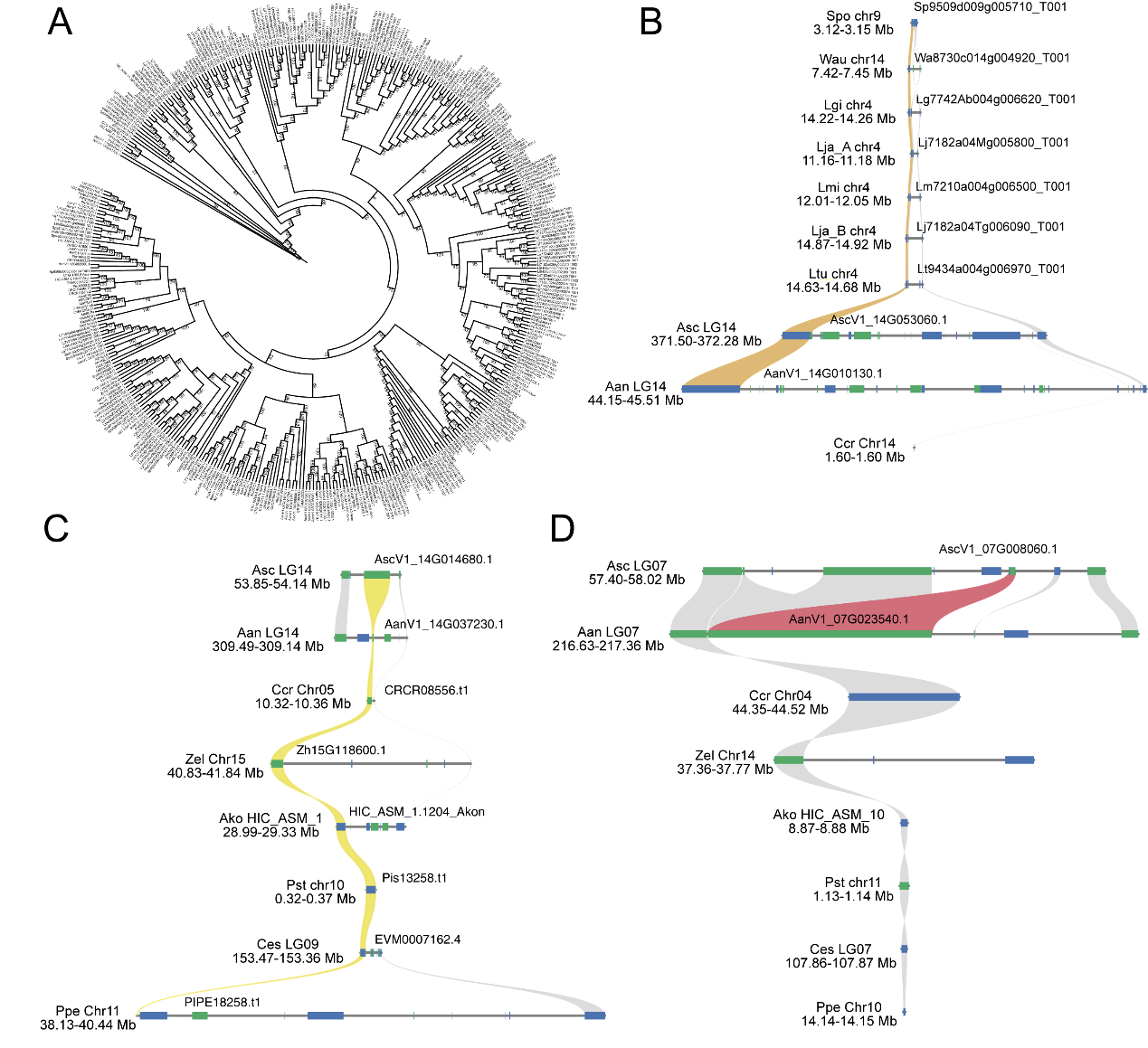
Figure S13. Phylogenetic and syntenic analyses of MADS-box genes in 14 Araceae.** (A) Maximum likelihood phylogenetic tree of the MADS-box gene family across 14 Araceae species, with *Arabidopsis* and *Oryza sativa* included as references. Bootstrap values from 1,000 replicates are shown at each node. (B) Synteny analysis of the *AGL6* gene among 14 Araceae species. *AGL6* is absent in Class III, which comprises *Cryptocoryne crispatula* (Ccr), *Zantedeschia elliottiana* (Zel), *Amorphophallus konjac* (Ako), *Pistia stratiotes* (Pst), *Colocasia esculenta* (Ces), and *Pinellia pedatisecta* (Ppe). (C–D) Synteny analysis of the *SOC1* gene among 14 Araceae species. *SOC1* is absent in Class I, which includes *Spirodela polyrhiza* (Spo), *Wolffia australiana* (Wau), and species of the genus *Lemna* (*L. minor* (Lmi), *L. japonica* (Lja), *L. turionifera* (Ltu), *L. gibba* (Lgi)). *A. andraeanum* ‘Alabama’ (Aan), *A. scherzerianum* ‘Red Lantern’ (Asc). Conserved collinear gene blocks are connected by colored ribbons, and focal genes are highlighted in color. Gene IDs and chromosomal positions are indicated.

**
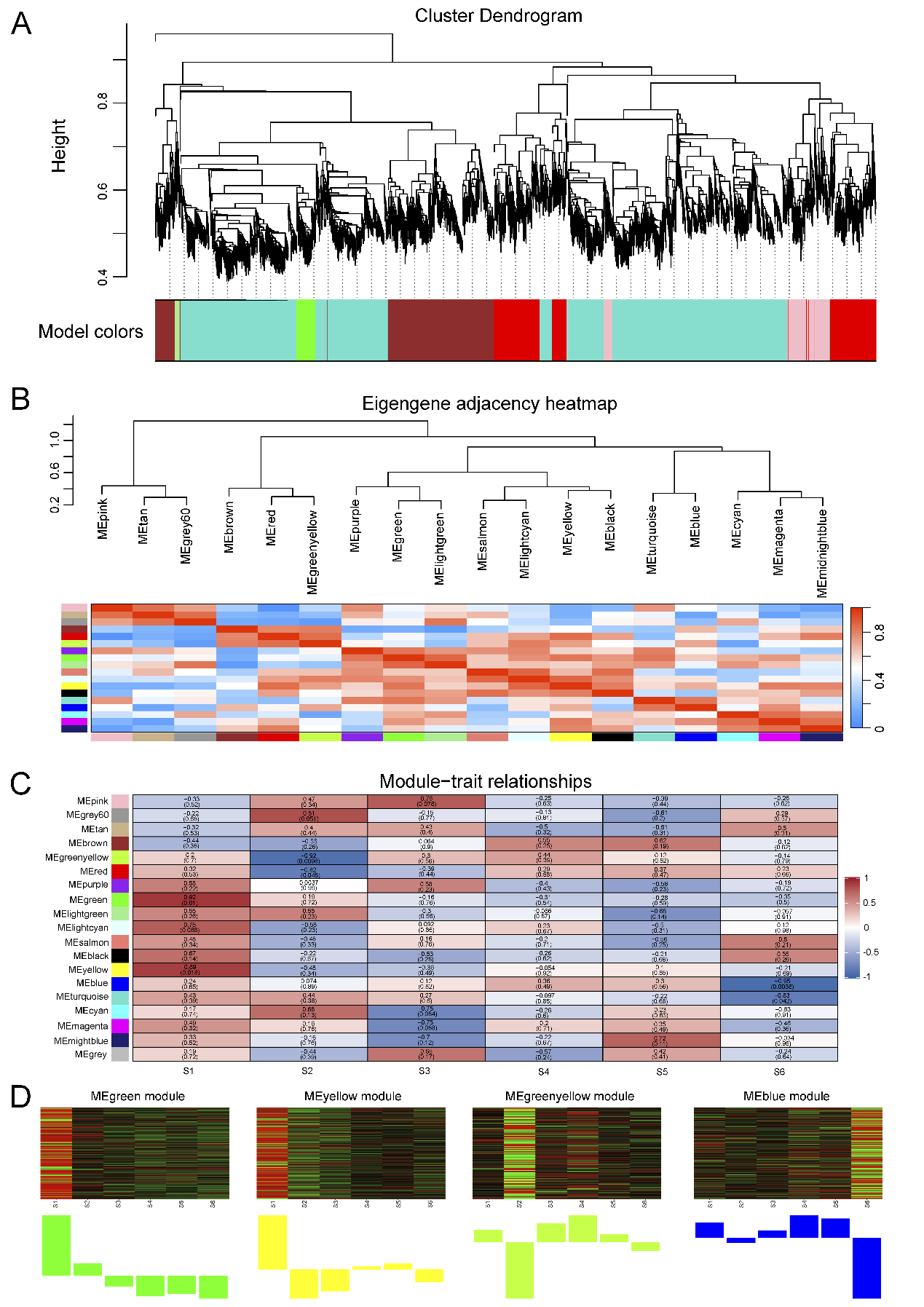
****Figure S14.** **Weighted gene co-expression network analysis of six spadix stages in *A. andraeanum* ‘Alabama’.** (A) Gene dendrogram and module assignment. (B) Visualization of the eigengene network representing the relationships among the modules and weights. (C) Module-trait associations. Each row corresponds to a module eigengene and each column corresponds to a volatile organic compound. Each cell contains the corresponding correlation and p-value. The table is color-coded by correlation according to the color legend. (D) Four modules that are significantly associated with three target compounds.

**
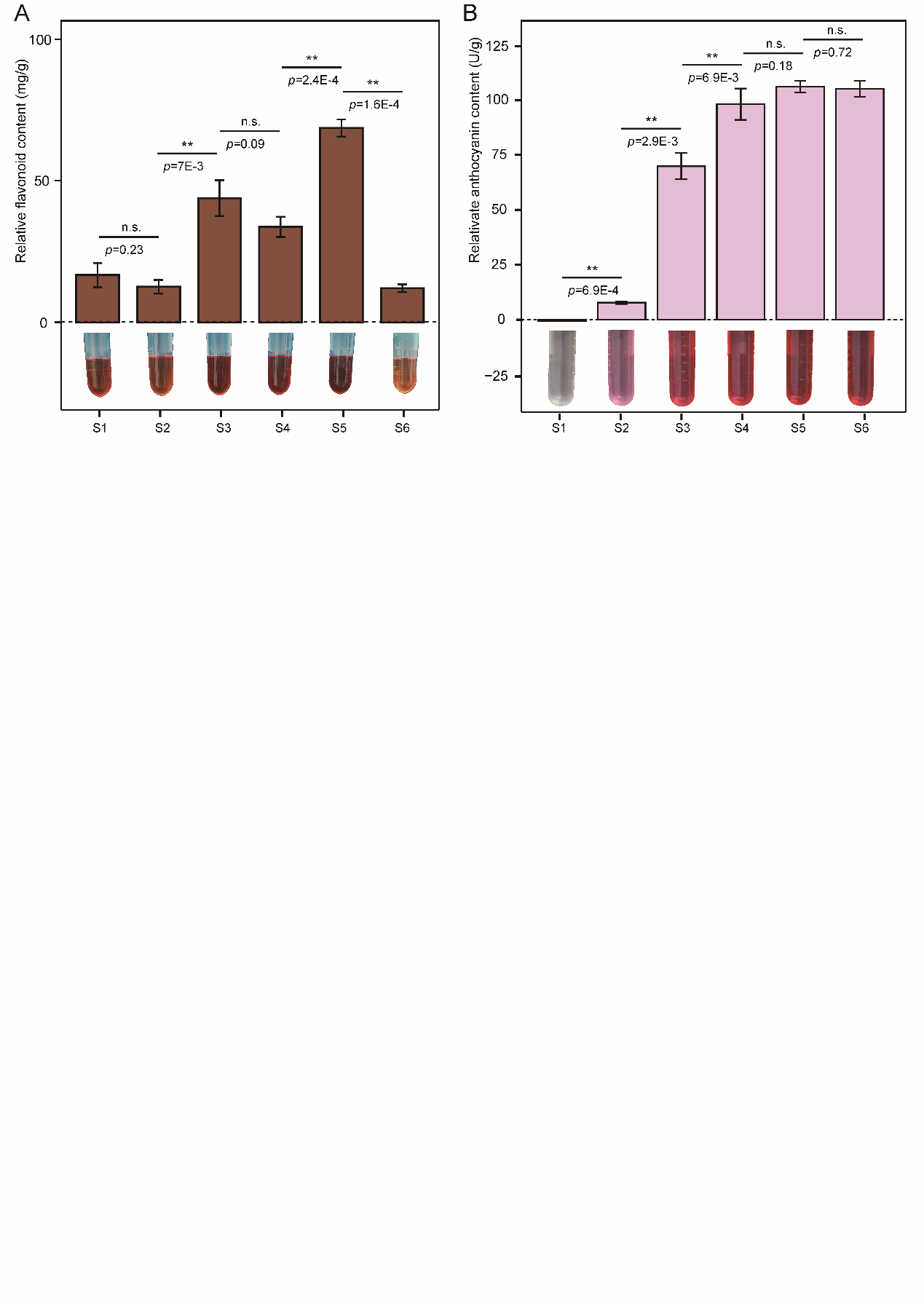
****Figure S15.** **Relative contents of flavonoids (A) and anthocyanins (B) in spathe of *A. andraeanum* ‘Alabama’ at six stages.**

**
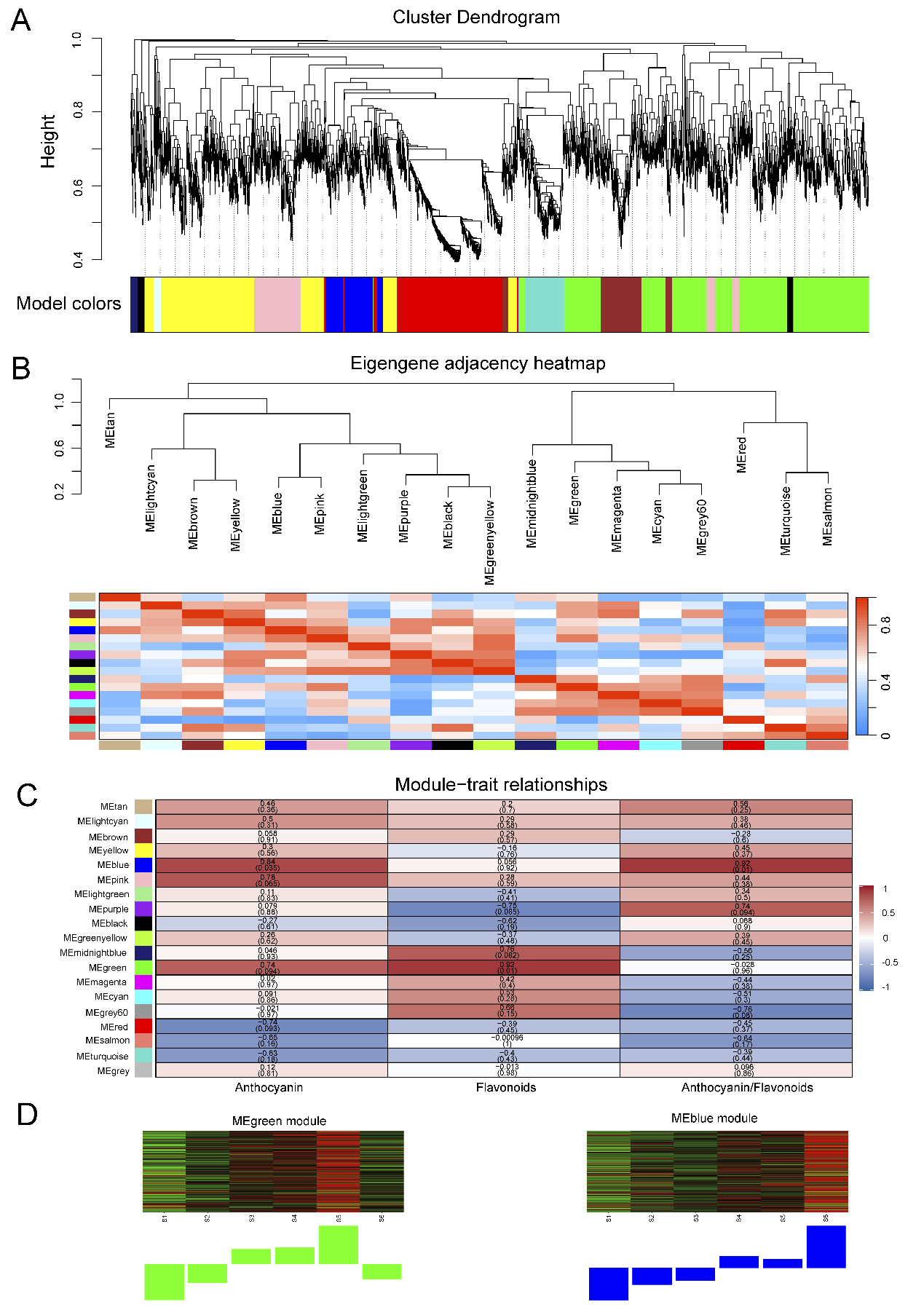
****Figure S16.** **Weighted gene co-expression network analysis of six spathe stages in *A. andraeanum* ‘Alabama’.** (A) Gene dendrogram and module assignment. (B) Visualization of the eigengene network representing the relationships among the modules and weights. (C) Module-trait associations. Each row corresponds to a module eigengene and each column corresponds to a volatile organic compound. Each cell contains the corresponding correlation and p-value. The table is color-coded by correlation according to the color legend. (D) Two modules that are significantly associated with three target compounds.
